# Multiple and Promising Applications of Strontium (Sr)-Containing Bioactive Glasses in Bone Tissue Engineering

**DOI:** 10.3389/fbioe.2019.00161

**Published:** 2019-07-05

**Authors:** Saeid Kargozar, Maziar Montazerian, Elisa Fiume, Francesco Baino

**Affiliations:** ^1^Tissue Engineering Research Group (TERG), Department of Anatomy and Cell Biology, School of Medicine, Mashhad University of Medical Sciences, Mashhad, Iran; ^2^Center for Research, Technology and Education in Vitreous Materials, Federal University of São Carlos, São Carlos, Brazil; ^3^Department of Applied Science and Technology, Institute of Materials Physics and Engineering, Politecnico di Torino, Turin, Italy; ^4^Interuniversity Center for the Promotion of the 3Rs Principles in Teaching and Research, Italy

**Keywords:** bioactive glasses, strontium, cement, coating, scaffold, osteogenesis, tissue engineering

## Abstract

Improving and accelerating bone repair still are partially unmet needs in bone regenerative therapies. In this regard, strontium (Sr)-containing bioactive glasses (BGs) are highly-promising materials to tackle this challenge. The positive impacts of Sr on the osteogenesis makes it routinely used in the form of strontium ranelate (SR) in the clinical setting, especially for patients suffering from osteoporosis. Therefore, a large number of silicate-, borate-, and phosphate-based BGs doped with Sr and produced in different shapes have been developed and characterized, in order to be used in the most advanced therapeutic strategies designed for the management of bone defects and injuries. Although the influence of Sr incorporation in the glass is debated regarding the obtained physicochemical and mechanical properties, the biological improvements have been found to be substantial both *in vitro* and *in vivo*. In the present study, we provide a comprehensive overview of Sr-containing glasses along with the current state of their clinical use. For this purpose, different types of Sr-doped BG systems are described, including composites, coatings and porous scaffolds, and their applications are discussed in the light of existing experimental data along with the significant challenges ahead.

## Introduction

Bioactive glasses (BGs) are currently used as implantable materials for the management of various types of bone disorders and diseases (Baino et al., 2018,a; Kargozar et al., 2019a; Miola et al., 2019). After five decades from the invention of Hench's 45S5 formulation, numerous commercially- produced BGs are being now used as effective substitute materials for hard tissue engineering. A few key advantages have been counted for BGs regarding their application in bone regeneration including the ability to bond to the living tissues and to improve the growth and proliferation of osteoblasts, the stimulation of osteogenesis and angiogenesis, and the local induction of antibacterial and antifungal effects (Kargozar et al., 2017, 2018a, 2019b,c; Mozafari et al., 2019). The main reason for these abilities is related to the release of various metallic ions (e.g., silicate ions, Cu^2+^) from the glass structure into the surrounding biological environment (Kargozar et al., 2018b). Therefore, the researchers incorporate different ions into the glass structure in order to obtain favorable biological effects. Indeed, the ionic dissolution products of BGs stimulate the expression and secretion of biochemical markers involved in the repair and regeneration of bone such as osteocalcin (OCN), osteopontin (OPN), and vascular endothelial growth factor (VEGF) (Jell and Stevens, 2006; Johari et al., 2016; Kargozar et al., 2019d).

Among the different therapeutic elements, increasing attention has been paid to add Sr^2+^ ions to silicate-based glasses for bone reconstruction application. Strontium (Sr) is an alkaline earth metal, which normally exists in the human skeleton (Hodges et al., 1950). This element can be substituted in the calcium (Ca) positions of apatite with a strong bone-seeking property (Vaughan, 1975). Sr in the form of strontium ranelate (SrR) has been used for the treatment of a common bone disease, i.e., osteoporosis, over the last decades (O'Donnell et al., 2006). Strontium has also been used in toothpaste to repair decayed teeth due to its restorative capability (Huang et al., 2016). Sr^2+^ ions via improving osteoblast activity and inhibiting osteoclast function can enhance the density of the bone tissue, resulting in a significant reduction in fracture risk in mammals (Bonnelye et al., 2008). This increase is supposed to be connected with the potential of Sr^2+^ ions to enhance the expression and activity of osteogenesis-related genes and proteins [e.g., Runx2 and alkaline phosphatase (ALP)] through the activation of a couple of cellular signaling pathways (Hurtel-Lemaire et al., 2009; Peng et al., 2009). Moreover, some researchers have proposed the immunomodulatory effects of Sr element, which provides an appropriate environment for enhancing bone regeneration (Zhang et al., 2016). It was reported that Sr^2+^ ions might have antibacterial property when released from different product formulations (Li et al., 2016; Liu et al., 2016). Having the above-mentioned characteristics, Sr is being currently used in various types of glasses (melt-derived and sol-gel) for bone tissue engineering applications. Moreover, there are several studies in which Sr-doped BGs have been added to polymeric matrices for the development of osteoinductive composites in order to accelerate the bone tissue reconstruction (Kargozar et al., 2018c).

Several formulations of glasses have been developed in which Ca is partially substituted by Sr. These formulations are presented as silicate-, phosphate- and borosilicate-based glasses (Lao et al., 2008; Abou Neel et al., 2009; Pan et al., 2009; Sriranganathan et al., 2016), which can be produced as fine powders, granules, fibers, and three-dimensional (3D) scaffolds (Ren et al., 2014; Kargozar et al., 2016; Yin et al., 2018; Baino et al., 2019).

In the present study, we aim to cover the main aspects around Sr-containing glass-derived biomaterials that are relevant to their application in bone tissue engineering strategies. The basic properties of Sr-doped glasses, such as their physicochemical characteristics and reactivity upon contact with biological fluids, are discussed and critically compared in section Sr-containing BGs: an overview. The use of these materials to prepare cements, coatings, and composites is described in section Cements, composites, coatings, and glass-ceramics based on Sr-doped BGs, while section Three-dimensional (3D) scaffolds is focused on Sr-based bioactive glass porous scaffolds. The results of *in vitro* and *in vivo* experiments are discussed in section Biological functions of strontium to show the great importance of Sr as a therapeutic ion for bone repair and regeneration. In this regard, Sr-regulated cell signaling pathways involved in osteogenesis induction, osteoclastogenesis, and bacterial inhibition are presented in detail to provide a comprehensive picture of the biological significance of Sr-doped BGs. A concise forecast for future research is then exposed in section Conclusions and outlook, just before the Conclusions. In summary, the present study aims to show a comprehensive view of synthesis methods, structure and reactivity, and biological outputs of this type of materials as an updated and focused study for researchers working in the field. While some general reviews have previously been published on bioactive glasses, including some contributions oriented to clinical applications and commercial products (Jones et al., 2016; Baino, 2017), to the best of the authors' knowledge this is the first review paper dealing specifically with Sr-doped BGs and related biomaterials.

## Sr-Containing BGs: an Overview

Sr-containing BGs have recently attracted much interest among researchers and scientists due to the positive effects of Sr on bone metabolism by preventing bone resorption and enhancing new tissue growth, both *in vitro* and *in vivo* (Hoppe et al., 2011). All these aspects, combined with the well-known features of BGs (Rahaman et al., 2011), make Sr-containing BGs highly appealing in the treatment of degenerative bone pathologies (e.g., osteoporosis) (Wei et al., 2014; Mao et al., 2017).

### Synthesis Methods: Melt-Derived and Sol-Gel Glasses

According to the specific needs related to the final clinical application, BGs can be produced either by traditional melt-quenching route or by sol-gel method, which can confer specific physical and chemical properties to the material, regardless of the composition (Fiume et al., 2018). In the past, the sol-gel route was demonstrated to allow better structural control and homogeneity of the final material compared to traditional melt-derived glasses. Moreover, the possibility to obtain a mesoporous structure often represents an advantage in bone tissue engineering applications because of the higher specific surface area of the final product (Zhang et al., 2016), which induces an acceleration of the hydroxyapatite (HA) formation kinetics on the surface of the glass by providing more reaction sites for the nucleation of the crystals (Baino et al., 2018,a).

In a typical melt-quenching approach, the melting of the reagents is carried out at high temperatures (in the typical range of 1,300 to 1,550°C) in electrical furnaces. Adequate processing parameters (i.e., heating rate and melting time) are fundamental to obtain a homogeneous and bubble-free melt, thus guarantying the high quality of the final product. According to the different desired applications, the melt could be cast into molds, quenched in water (“frit”) or drawn into fibers (Ylanen, 2011; Fagerlund and Hupa, 2016).

Sol-gel process is defined as a chemical-based processing technique for the production of ceramic materials at noticeably lower processing temperatures since the polymerization reaction of a solution containing precursors occurs at room temperature resulting in the formation of the 3D network, which are properly chosen in order to tailor the final composition of the system to the intended purpose (Hench and West, 1990). Briefly, sol-gel synthesis allows the production of ceramic materials by three steps: (i) preparation of the precursor solution (sol), (ii) gelation process of the prepared sol, and (iii) removal of the solvent by thermal treatments. Doping glasses by the introduction of trace elements in the sol is relatively easier compared to the traditional melt-quenching route and allows preserving the bioactivity of the system while providing a specific therapeutic effect upon ion release (e.g., angiogenesis, antibacterial and antioxidant properties) (Owens et al., 2016).

As almost all the BGs compositions, also Sr-doped BGs can be produced either by melt-quenching route or sol-gel process, using, respectively, strontium carbonate or strontium nitrate as a precursor of SrO. Carbonates, in fact, are not of common usage in the sol-gel process since the temperatures used to stabilize the network (calcination process) would not be high enough for allowing the complete removal of undesired carbon residues.

The final products obtained by sol-gel method, including monoliths, porous scaffolds, fibers, coatings, and granules, are all characterized by the presence of a mesoporous texture, which is inherent of sol-gel materials (Owens et al., 2016; Baino et al., 2018). It is worth mentioning that sol-gel process was also widely used to synthesize spherical nanoparticles (Kargozar and Mozafari, 2018; Leite et al., 2018) to be used as nanocarriers for controlled drug release for the treatment of bone pathologies, such as osteoporosis (Fiorilli et al., 2018).

**Figure 1:**
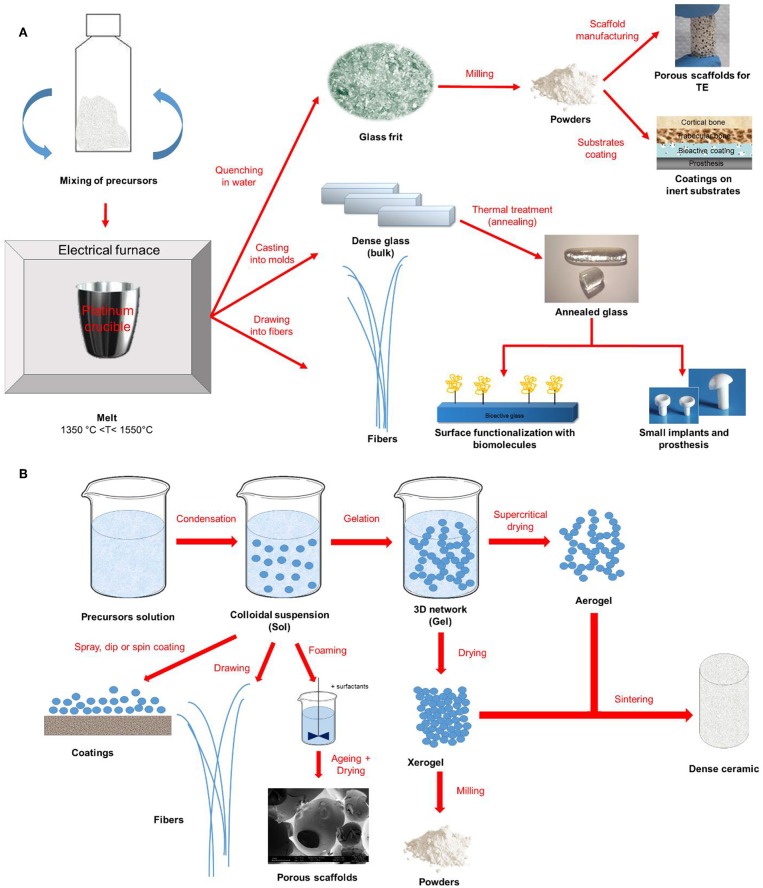
Schematic representation of **(A)** melt-quenching and **(B)** sol-gel route for bioactive glass synthesis and final products.

Figure 1 provides a schematic representation of the two synthesis approaches previously described together with some examples of final products that could be obtained.

### Thermal Properties, Crystallization, and Sintering Behavior of Sr-doped Bioactive Glasses

One of the major issues concerning the processing of BGs to obtain, for example, porous scaffolds, or coatings is the devitrification, which occurs upon the sintering treatments required for the densification of the structure. This is an aspect of concern in tissue engineering applications because the nucleation of crystalline phases, as well as the specific surface area, are directly linked to the reactivity of the surface itself and, thus, to the capability of the material to bond to the host tissue.

As a result, understanding the sintering conditions of glass powders and how the introduction of modifier cations in the glass network could affect the thermal properties and crystallization of the materials are among the key aspects to be considered when choosing a BG to fabricate sintered products for tissue engineering applications.

When introduced within the glassy matrix as a network modifier, SrO was found to affect the thermal behavior, sintering ability, microstructure and crystallization of the system, depending on both on the synthesis method and on the composition.

During the last decade, the thermal properties of Sr-doped bioactive glasses, together with crystallization kinetics and sinterability have been the object of study of several research groups.

**Figure 2:**
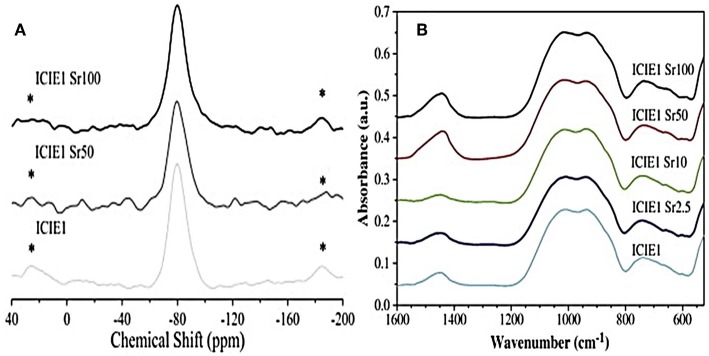
^29^Si MAS-NMR **(A)** and FTIR **(B)** spectra related to glasses with various calcium-to-strontium substitutions. The results reveal no significant effects on structural properties and network connectivity (Fredholm et al., 2010). *represents spinning sideband.

Sr^2+^ is often introduced in the glass network as a modifier cation by partially replacing CaO with SrO. Due to the similar chemical role played by the two oxides, no significant structural alterations of the network (Network Connectivity - NC) were reported, with predominant Q^2^ silicate structure (Figure 2).

The very similar field strength of Ca^2+^ and Sr^2+^ ions resulted in no significant shielding/de-shielding of the ^29^Si nuclei (Fredholm et al., 2010). Anyway, the larger size of the Sr^2+^ ion compared to that of Ca^2+^ was found to expand and weaken the network, thus leading to a modification of the thermal properties of the glass (Lotfibakhshaiesh et al., 2010; Goel et al., 2011; Hasan et al., 2015; Li et al., 2015; Bellucci et al., 2017).

A careful analysis of the currently-available literature suggests that the effect of the inclusion of Sr on the structural alterations of BGs is strongly affected by the use of mole or weight percent in the compositional design. When weight percentage (wt %) is used, the higher molecular weight (mol%) of SrO compared to CaO can determine an increase of silica content in mole percentage, thereby resulting in higher network connectivity. On the contrary, when the mole percentage is used, the opposite effect could be observed in melt-derived glasses (O'donnell and Hill, 2010).

Several thermal studies performed on melt-derived Sr-doped BGs revealed the possibility to enhance the densification of the structure upon sintering by increasing the SrO/CaO molar ratio while preserving the amorphous nature of the system.

DTA analyses performed by Goel et al., for example, reported no alterations in the glass transition temperature (T_g_) and increased values of the crystallization onset (T_c_) and the crystallization peak (T_p_) as a result of the increase in the Sr content up to 8 mol%, resulting in a significantly wider processing window (PW) of the glass, defined as the difference between the T_c_ and the T_g_ (Goel et al., 2011).

The complete thermal behavior and sintering ability of CaO-rich silica-based BGs modified by the replacement of CaO (10 mol%) with MgO, SrO or both in a 1:1 ratio was recently reported by Bellucci et al. (2017). Interestingly, DTA curves showed lower T_g_ of the modified glasses compared to that of the original composition (Bellucci et al., 2017). A similar outcome was found in another study by Lotfibakhshaiesh et al. (2010), who attributed the decrease of the T_g_ to the expansion of the glass network resulting from the replacement of Ca with Sr.

On the other hand, the substitution of Sr^2+^ for Na^+^, was found to induce an increase of the T_g_ from 591 to 760°C with (Li et al., 2015), despite no significant changes of the glass structure were detected by X-ray photoelectron spectroscopy (XPS), Raman Spectroscopy and MAS-NMR. In fact, even if both Sr^2+^ and Na^+^ ions act as network modifiers, the increase in the T_g_ could be attributed to the different valence of the two ions. In fact, while Na^+^ can compensate just one non-bonding oxygen NBO^−^, bivalent Sr^2+^, can charge compensate 2NBO^−^ at the same time.

One of the major differences between Sr-doped sol-gel and melt-derived glasses concerns the possibility to retain the amorphous structure of the material upon sintering. In sol-gel BGs, XRD analyses revealed that, as the Sr^2+^ increased within the composition, the tendency toward crystallization was more pronounced. In particular, a complete crystalline structure was observed when CaO was totally substituted by SrO, with diffraction peaks associated with the Sr_2_SiO_4_ phase (Taherkhani and Moztarzadeh, 2016). The presence of Sr-containing crystalline phases in sol-gel materials was already reported by Solgi and coworkers (Solgi et al., 2017), while no crystallization was observed in melt-derived glasses as a result of the increase in Sr content (Hill et al., 2004; Fredholm et al., 2010; Kargozar et al., 2016). In fact, most of the XRD patterns in melt-derived systems (Hill et al., 2004; Kargozar et al., 2016) revealed just a slight shift in the amorphous scattering maxima to lower 2θ-values as a result of the replacement of Ca^2+^ with Sr^2+^ ions because of the larger size of the Sr^2+^ ions than Ca^2+^ ions (Kargozar et al., 2016).

However, Massera et al. reported DTA analyses on phosphate melt-derived glasses (Massera et al., 2013), showed a shift of the main crystallization peak toward lower temperatures as a result of the increase of SrO within the composition up to 5 mol%. For SrO amounts higher than 10 mol%, however, a shift toward higher temperatures could be observed, and a second crystallization peak could appear (Massera et al., 2013; Dessou et al., 2017).

Even if a direct comparison between these studies is difficult to establish due to the different compositions of the systems investigated, it can be stated that synthesis method indeed plays a crucial role in defining the physical and thermal properties of Sr-doped BGs. It is believed that further studies focused on the direct comparison of equivalent compositions produced by the traditional melt-quenching route and sol-gel chemical synthesis would provide a valuable contribution in shedding light on these peculiar aspects.

### Structure and Reactivity of Sr-Doped Silicate, Borate, and Phosphate Glasses

Achieving highly-controlled ion release from biomaterials is one of the most important challenges in bone regeneration (Hoppe et al., 2011). In the last years, BGs have received much attention because of their interesting capability to promote cell attachment, proliferation, and differentiation thanks to their dissolution in a physiological environment based on an ion-release mechanism (Fiume et al., 2018).

The bioactivity mechanism, proposed for the first time by Larry Hench, is still accepted for silica-based BGs (Hench, 2006). Upon contact with biological fluids, the formation of a hydroxycarbonate apatite (HCA) layer is observed on the material surface, following the crystallization of the amorphous calcium phosphate film; at the same time, the ionic dissolution products released from the glass surface (basically calcium and silicate ions) confers osteogenetic properties to the material, stimulating the beneficial response of the surrounding tissue, which culminates in the mineralization of the newly-synthesized extracellular matrix (ECM) (Hench, 2006; Rahaman et al., 2011; Jones, 2015).

Probably, one of the most attractive aspects of BGs is the possibility to tailor their properties by introducing selected cations able to play a specific functional and/or biological role. In fact, it has been demonstrated that the addition of such cations, e.g., Sr^2+^, into the glass network, affect crystallization kinetics, crystallinity, and thermal stability of the system to devitrification, as already discussed in the previous section. Moreover, the formation of the surface HCA layer, observed during the bioactive mechanism of glass dissolution, and the osteogenetic properties were found to be deeply related both to the type of former oxides and to the presence of dopants.

According to the final application of the material, either slow or fast dissolution rates might be required. In fact, while higher dissolution rates are usually associated with high reactivity and enhanced capability to bond to the host tissue providing early-stage stability of the implant, it should be pointed out that having slow dissolution rates of the material could represent an advantage in those bone-repair applications characterized by weak metabolic activity typical of some pathologies, as slower reaction kinetics would be more suitable in order to match the physiological healing time of the tissue.

In the present section, the effect of Sr incorporation within the glass network of silicate, borate and phosphate glasses will be discussed by focusing the attention on the structure modification and the influence on the mechanism of reactivity with biological fluids (Oudadesse et al., 2011; Li et al., 2014).

#### Silicate Sr-doped BGs

As regards silicate Sr-doped bioactive glasses, several studies confirmed that the introduction of Sr as a network modifier was able to induce important modifications in the bioactivity rate based on both the composition design (O'donnell and Hill, 2010) and the synthesis method. pH measurements during bioactivity tests in SBF and XRD patterns revealed that Sr for Ca substitution in molar proportions in melt-derived systems was able to increase both the degradation rate and the apatite-forming ability of the system because of the expansion of the glass network determined by the larger dimensions of the Sr^2+^ ion compared to Ca one (Fredholm et al., 2011). In particular, Ca release was found to follow a linear trend for Sr substitution ≤10 mol%, but an increase of Ca release was observed for Sr substitution of 2.5 mol% because of the larger ionic radius of Sr element (1.16 A) in comparison to Ca (0.94 A), which expands the glass network. XRD pattern was characterized by the presence of apatite nucleation peaks that become more pronounced as the Sr content increases, while in the Sr-free control no apatite peaks were detected (Fredholm et al., 2011).

Interestingly, an opposite behavior was observed in sol-gel glasses, where the replacement of Sr with Ca in the glass composition was proved to slow down the formation of the apatite layer on to the glass surface (Hesaraki et al., 2010a; Lao et al., 2013). A similar trend was found in sol-gel derived Sr-containing 13–93 nanoparticles doped with different amounts of Sr (Hoppe et al., 2014). Interestingly, at the nanoscale, an inhibitory effect of Sr-doping on the crystallization of the deposited calcium phosphate layer with respect to the Sr-free 13–93 nano-BG was observed because of the incorporation of Sr in the HA layer. This can be supported by Aina et al. (2013) study, showing a decrease in crystallite size and degree of crystallinity after introducing Sr into HA due to the larger size of Sr^2+^ ion in comparison to Ca, which causes an increase in d-spacing and crystal cell unit parameters. Therefore, the high local release of Sr^2+^ ions facilitated by the high surface area of sol-gel materials might have led to inhibited HA crystallization. These outcomes are in line with Rokidi and Koutsoukos's findings (Rokidi and Koutsoukos, 2012), showing that the presence of strontium in supersaturated solutions of calcium phosphate retarded the crystal growth of both octacalcium phosphate and HA.

Anyway, despite the dissolution rate and HA nucleation kinetics could be delayed as a result of the increase in Sr content which can increase the chemical stability of the glass, all the studies proved the possibility to retain the bioactive potential of the systems upon Sr-doping in different amounts.

#### Borate Sr-Doped BGs

Besides silicate BGs, borate systems recently gained increased scientific interest as attractive materials for several biomedical applications. Unlike the case of silicon, the coordination number of boron does not allow the formation of fully three-dimensional structures, which results, from a chemical viewpoint, in a lower resistance of network interconnection and hence in higher degradation rates during contact with body fluids (Wright et al., 2010). As a result, the cytotoxicity deriving from the rapid release of boron in the physiological environment has to be carefully controlled (Balasubramanian et al., 2018). The incorporation of Sr and other modifiers within the network of borate BGs seems to be one of the most effective strategies used to face this issue. The effect of different bivalent modifier oxides (i.e., BaO, SrO, ZnO, and MgO) on melt-derived borate-based glasses was recently investigated in a study by Kumari et al. (2017). MgO, SrO and BaO are conventional modifiers that enter the glass network by disrupting B–O–B, P–O–P and B–O–P bonds. In particular, the addition of modifying oxides in borate glasses can augment the network via the following events: (i) breaking of B–O–B bonds with the contiguous creation of non-bridging oxygens; (ii) increasing the oxygen coordination of boron; and (iii) combining both mechanisms mentioned above (Hasan et al., 2015).

In borosilicate and borate glasses, a slower release of boron was observed as a result of the increase in strontium content. The ion movements within the network are, in fact, partially inhibited by the expansion of the network caused by the larger size of Sr^2+^ ion (Pan et al., 2009). Moreover, while Na and B are easily released into the external environment, SrO uses to form Sr (OH)_2_ species, which are chemically more resistant and difficult to dissolve (Hasan et al., 2015).

#### Phosphate Sr-doped BGs

Phosphate-based BGs are typically used in those clinical applications which require high dissolution rates of the implant (Brow, 2000).

In 2017, Patel and coworkers used Sr/Ca substitution to control and tune the dissolution behavior of melt-derived phosphate glasses in the 40P_2_O_5_−(16−x)CaO−20Na_2_O−24 MgO–x SrO system (x = 0, 4, 8, 12, 16 mol%) in order to achieve an accurate control on the rate of release of therapeutic ions included within the glass composition. The initial addition of SrO into the glass composition (up to 4 mol%) resulted in a decrease of the dissolution rate of the glass, thus suggesting an increase of the cross-linking between phosphate chains (Patel et al., 2017).

Kapoor and coworkers studied the structure-properties relationships in different melt-derived alkali-free phospho-silicate glass compositions co-doped with Zn^2+^ and Sr^2+^ ions (Kapoor et al., 2014). In particular, the attention was focused on the co-doping effects of Sr and Zn on the chemical dissolution behavior and bioactive mechanism in SBF. No effect of the Zn/Mg and Ca/Sr substitution was observed on the NC, which remained constant at about 1.95. Despite the lower NC compared to that of commercial 45S5 Bioglass®, the system showed lower solubility as a result of the ionic field strength associated with its constituent ions. There was a significant difference in the leaching of Zn^2+^ and Sr^2+^ ions in SBF and Tris-HCl despite equimolar ZnO, and SrO concentrations were incorporated, with a higher rate of release for Sr (Kapoor et al., 2014).

Phosphate glass properties make them very appealing as basic materials for the production of resorbable implants. In this attempt, melt-derived Sr-containing polyphosphate glasses doped with Mg and Ti were investigated by Weiss et al. (2014). The inclusion of Mg and Ti was found to increase the bonding strength between phosphate chains resulting in a higher glass stiffness, better mechanical properties, and lower degradation rates in Tris-HCl solution. The HA layer observed after 15 days of immersion in SBF was thicker and denser for the doped systems, thus suggesting a stimulatory and synergic effect of multiple ions on the bioactivity mechanism of the glass (Weiss et al., 2014).

Interestingly, some studies demonstrated that the improvement in the chemical stability of phosphate glasses seems to be not affected by the thermal properties (Hesaraki et al., 2010b; Stefanic et al., 2018).

Stefanic et al. investigated the effect of Sr substitution in 40P_2_O_5_-25CaO−5Na_2_O–(30 – x) MgO–xSrO systems (x = 0, 1, 5, 10 mol%). FTIR analysis revealed no significant structural modifications with no variations in the O/P ratio and the Q speciation. However, phosphate bands shifted toward lower wavenumbers as the Sr content increased from 1 to 10 mol%. This shift could be attributed to the lower field strength of Sr^2+^ ions, which have higher atomic number compared to that of Mg^2+^ ions, as the total divalent cation-to-phosphate ratio did not change within all the glasses investigated. Despite the higher thermal stability of the Sr-free system, the chemical durability of these melt-derived glasses in water was found to decrease with decreasing Sr content, and it was characterized by linear degradation and highly controllable profiles (Stefanic et al., 2018).

#### Comparative Remarks

Table 1 provides a summary of the literature results discussed in the previous sections and relates thermal and structural properties to the bioactive potential of the systems analyzed. In summary, it can be stated that there is a strong dependence on the basic compositional system, and some peculiar trends can be observed. While Sr doping in silicate glasses was found to enhance the mechanism of bioactivity and accelerate the ion release rates, the increase of SrO in borate and phosphate systems typically led to improved chemical stability of the material. However, it is worth pointing out the presence of some exceptional cases (reported in Table E1) that suggest caution in generalizing the results. In fact, a direct comparison between different systems is hard to carry out since all the properties of glasses are affected by multiple factors that simultaneously contribute to the final and complete behavior of the examined material.

**Table 1:** Comparison among the Sr-doped glass systems discussed in the section Sr-containing BGs: an overview.

**Glass system**	**Synthesis method/former oxide**	**The object of the study**	**Thermal and structural properties**	**Bioactivity tests**	**References**
Na_2_O/K_2_O/MgO/CaO/B_2_O_3_ SiO_2_/P_2_O_5_/SrO	M/B	Controlled release of borate and Sr^2+^ ions for new bone formation	-No crystallization upon doping -No changes in the glass structure	-Complete conversion to apatite -Controlled degradation and ion release below the cytotoxic level	Li et al., 2016
CaO/SrO/SiO_2_/MgO/P_2_O_5_ /CaF_2_	M/S	Effect of Sr for Ca substitution on structural features, sintering behavior, and apatite-forming ability	- No changes in the glass structure - Wider PW up to 10 SrO mol%	-Lower apatite-forming ability in SBF - Lower chemical degradation in TRIS-HCl -Ion release within therapeutically effective range	Kargozar et al., 2016
Na_2_O/SrO/SiO_2_/TiO_2_/CaO	M/S	Influence of Na^+^ and Sr^2+^ on solubility	- No changes in the glass structure - Higher T_g_	- Lower ion release rates	Ren et al., 2014
B_2_O_3_/SrO/TiO2 B_2_O_3_/SrO/Na_2_O/TiO_2_	M/B	Production of a borate glass system without the addition of other network formers; assessment of the physical, structural, thermal, and biological properties	-Higher T_g_ -Higher glass density	- SrO content influences degradation rate and ion release -Sr concentration above cytotoxicity levels	Yin et al., 2018
CaO/SrO/SiO_2_/P_2_O_5_/Na_2_O	M/S	Influence of Sr for Ca substitution on physical properties	- No changes in the glass structure - Higher glass density - Lower oxygen density (network expansion) - Lower dilatometric softening point - Higher thermal expansion coefficient - Lower T_g_	_	Baino et al., 2019
CaO/ SrO-MgO/SiO_2_/Na_2_O K_2_O/P_2_O_5_	M/S	Combination of the thermal behavior of Ca-rich silicate glasses with an improvement in biological results of MgO- and SrO-modified glasses	- Improved thermal stability - Improved mechanical properties	- Strong apatite-forming ability	Jones et al., 2016
CaO/SrO/SiO_2_/MgO/Na_2_O K_2_O/ZnO/P_2_O_5_	M/S	Influence of Sr/Ca substitution on the sintering behavior	- Lower T_g_ - Higher T_c_ - Wider PW	_	Baino, 2017
SiO_2_/CaO/SrO	SG/S	Development of Sr-delivering glasses	- No alterations in the mesoporous texture	- Enhanced bioactivity - Increased reaction kinetic	Wei et al., 2014
SiO_2_/CaO/MgO/SrO	SG/S	Synthesis, characterization, and investigation of the apatite-forming ability in SBF	- Crystalline phases (calcium and strontium silicates)	- Good apatite-forming ability - Apatite layer after 3-5 days immersion in SBF	Fiume et al., 2018
CaO/SrO/P_2_O_5_/Na_2_/CaO/SrO	M/P	Glass fiber production	- Higher thermal stability - Wider PW	Reduced phosphate ions release - Formation of the apatite layer - SrO and MgO embedded in the apatite layer - Improved chemical stability	Baino et al., 2018,a
P_2_O_5_/CaO/SrO/Na_2_O/MgO SrO	M/P	Investigation of phosphate glass formulation for controlled Sr release	- Lower T_g_ and T_m_ - Broadening of the main crystallization peak - No changes in the glass structure	- Higher chemical durability - Lower dissolution rates	Hench, 2006
SiO_2_/P_2_O_5_/CaO/SrO	SG/S-P	Production and characterization of Sr-doped silico-phosphate glasses	- Higher T_p_ - Nucleation of new crystalline phases - Increase in the gel viscosity	- Higher biodegradation rate	Hesaraki et al., 2010b

M, Melt-quenching route; SG, Sol-gel route; S, Silicate glasses; B, Borate glasses; P, Phosphate glasses; PW, Processing Window; T_g_, Glass transition temperature; T_c_, Crystallization onset temperature; T_p_, Maximum rate of crystallization temperature; T_m_, Melting temperature; SBF, Simulated body fluid. All the results are referred to the doped system, compared to the undoped one. If the effect of SrO replacement for another oxide is specifically investigated, the oxide couple (e.g., SrO/CaO) is indicated in the column “Glass system.”

### Atomistic Simulations

Understanding the relationship that exists between glass structure and functional properties is not always immediate, especially when the design of the glass composition becomes complex and rich of different elements whose effects are the result of the interaction of multiple factors.

In this section, atomistic simulations will be presented as a valuable instrument aimed at rationally designing glass compositions in order to define, investigate and better understand the structure, dissolution, and bioactivity mechanisms of glasses used in biomedical applications thanks to the rapid increase in computing power and development of new algorithms and methodologies (Tilocca, 2010; Xiang and Du, 2011; Du and Xiang, 2012).

**Figure 3:**
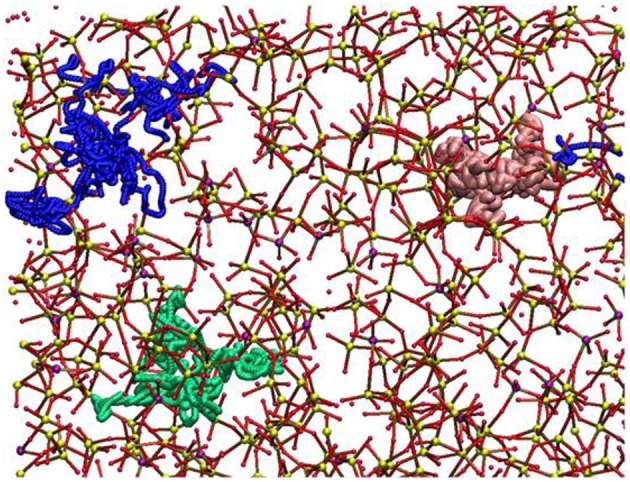
Diffusion pathways suggested for atoms of Na (green ball), Ca (blue ball), and Sr (pink ball). The other elements are depicted as Si (small yellow ball), P (small purple ball), O (small red ball) (Du and Xiang, 2012).

Molecular dynamics (MD) simulations are currently the most widely used method that allows very accurate and reliable glass structural models to be generated. Experimental studies showed that Sr-containing BGs, especially in the case of SrO/CaO substitution, did not exhibit very large modifications in terms of glass structure because of the similarity of Sr^2+^ and Ca^2+^ ions from a chemical viewpoint. Apart from these preliminary considerations, MD simulations were indeed useful to define the local environment and the diffusion behavior of SrO containing BGs (Xiang and Du, 2011; Du and Xiang, 2012; Xiang et al., 2013). The effects of SrO/CaO substitution on glass diffusion and bioactivity was deeply investigated by Du and Xiang (2012), Du and Xiang (2016) on three different compositions characterized by a silica content ranging from 46 to 65 mol% to cover multiple bioactivity levels (Du and Xiang, 2016). The local environment around modifiers cations, as well as network connectivity, were determined as a function of the glass composition. Figure 3 shows the diffusion pathways of Na, Ca, and Sr obtained by MD simulations.

Sodium ions were found to have a higher diffusion coefficient and lower energy barriers compared to Ca^2+^ and Sr^2+^ ions. Interestingly, an increase in silica content led to a decrease in the diffusion coefficient of the modifiers cations.

Because of the similarities of the self-diffusion coefficients and energy barriers of Sr^2+^ and Ca^2+^, Du and Xiang suggested that it is actually possible to maintain almost unaltered the basic physicochemical properties of the starting glass composition while enhancing tissue growth due to the release of Sr^2+^ ions (Du and Xiang, 2016).

While in classical MD simulations the interatomic forces are approximated by an empirical potential, easier to compute but affected by the introduction of approximations and loss of information on the electronic structure, in a recent study (Christie and de Leeuw, 2017). They investigated the effect of Sr addition on the bioactivity of phosphate glasses by developing a new interatomic potential able to consider the polarizability of oxygen ions in order to investigate the medium-range structure, which is responsible for the bioactivity mechanism. Several Sr-containing glass compositions were simulated by both classical and first-principle MD. In general, it was confirmed that Sr incorporation caused minimal changes in the dissolution of the glass, and the bioactivity remains preserved. The NC and Q^n^ distribution were shown to be essentially unaffected by the incorporation of Sr. The first result was to be expected, as the network connectivity depends on the ratio of the number of oxygen atoms to the number of phosphorus atoms, which does not change for SrO/CaO or SrO/Na_2_O substitutions. The Q^n^ distribution might change, but the amounts of Sr incorporated in the studied compositions were relatively small (10 mol% max.) and, thus, the associated change was small, too (Christie and de Leeuw, 2017).

## Cements, Composites, Coatings, and Glass-Ceramics Based on Sr-doped BGs

The majority of the BGs form a strong interfacial bond with the bone (Hench, 2013). An initial investigation by Piotrowski et al. (1975) proved that the interfacial bond between 45S5 Bioglass® and cortical bone in rat and monkey models is equal to or even higher than the strength of the host bone (Piotrowski et al., 1975), and this finding was eventually confirmed clinically (Montazerian and Zanotto, 2017a). As summarized in the previous section, many Sr-doped BGs have excellent biochemical compatibility; however, they have some limitations from a biomechanical viewpoint. The bending strength and fracture toughness of most of the compositions discussed above are in the range of 40–60 MPa and 0.5–1 MPa m^1/2^, respectively. These values are <50–150 MPa and 2–12 MPa m^1/2^, which are the typical ranges of cortical bone (Hench, 1991), and thus make Sr-doped BGs inappropriate for load-bearing applications. However, for some applications, low strength and fracture toughness are offset by the low elastic modulus of the glass (30–35 GPa), which is close to that of cortical bone (7–30 GPa). Therefore, the low strength does not question the usefulness of Sr-doped BGs for several important applications like cements, composites, and coatings. Low strength also does not affect the application of BGs as buried, low-loaded and compressively loaded implants, or in the form of powders and a bioactive phase in bone cements. Thus, several Sr-doped BGs were considered for the development of coating or composite to expand their range of applications. Furthermore, the development of glass-ceramics (GCs) provides another option for improving the mechanical properties of BGs.

### Bone Cements

Conventional polymeric bone cements like poly(methyl methacrylate) (PMMA) and glass ionomer cements are among the most used materials in dentistry and orthopedics, but they have numerous drawbacks. They commonly show poor bonding with bone, a high exothermic reaction *in situ*, low mechanical reliability, and inadequate radiopacity. Moreover, the high-temperature or elaborated processing techniques, slow degradation rate, and low strength are other limitations of commercial ceramic bone cements such as calcium phosphate cement (CPC) and HA (Kenny and Buggy, 2003). Therefore, the objective of numerous studies was to develop and characterize bone cements composed of reinforcing components like Sr-doped BG particles that serve as the reinforcing and radiopaque phase in polymers or sintering aid in ceramics. In addition, the Sr-doped BGs are usually selected due to their outstanding characteristics, including bioactivity, osteogenic potential, and the capability of the controlled release of Sr^2+^ ions.

A Sr-doped HA bone cement was prepared to enhance bioactivity and biocompatibility. The release of Sr^2+^ ions was supposed to the main reason for promoted osteoblast proliferation, which could facilitate the precipitation of newly-formed HA and resulting in the enhanced strength of the bone-cement interface (Cheung et al., 2005; Ni et al., 2006).

The preparation of an injectable Sr-containing CPC has been successfully reported with promising properties, including setting time, compressive strength, and radiopacity (Yu et al., 2009). This cement could improve the proliferation and differentiation of both osteoblastic cells and human bone marrow mesenchymal stem cells (hBMSCs) *in vitro* (Kuang et al., 2012; Schumacher et al., 2013) and the new bone formation was accelerated at the bone-cement interface, as well as in the entire metaphyseal fracture defect site in ovariectomized rats (Thormann et al., 2013).

#### Sr-BGs in Ionomer Cements

Starting from 2008, glass polyalkenoate cements (GPCs), used for restorative purposes in dentistry and orthopedics, were the subject of extensive research by Boyd et al. (2008), Wren et al. (2008), Clarkin et al. (2009), Wren et al. (2010), Wren et al. (2013). They employed new SiO_2_-ZnO–CaO–SrO glasses instead of the commercial fluoro-aluminosilicate glass. The glass usually reacts with an aqueous portion of the cement such as polyacrylic acid (PAA). The degradation of the glass structure is regulated by PAA, leading to the release of metallic cations into the aqueous phase of the setting cement. The carboxylate groups cross-link these cations on the PAA chains; embedding reacted and unreacted glass particles in a hydrated polysalt matrix of the cement (Boyd et al., 2008; Wren et al., 2008, 2010, 2013; Clarkin et al., 2009).

Different from aluminum as a neurotoxin, zinc is expected to inference positively the proper functioning of the immune system and to impart antibacterial properties to the cement (Kargozar et al., 2018a). Sr^2+^ ions were substituted with Ca^2+^ in the glass because their ionic radii are similar. Furthermore, strontium was known to have a lot of beneficial effects on bone, to share some of the same physiological pathways as Ca and to improve the radiopacity.

Boyd et al. (2008) produced GPCs from 48SiO_2_−36ZnO–(16-x)CaO–xSrO (x = 0, 4, 8, and 12 mol%) glasses and PAA. Glass frits were prepared via the conventional method of melting-quenching in water. They prepared the cements by thoroughly mixing 2 g of glass (particle sizes < 45 μm) with 0.6 g of PAA powders and 0.9 mL of distilled water on a glass plate. Thorough mixing of cements was undertaken during 30 s. The results reported in this research indicated that the replacement of Ca with Sr in the glasses has no significant influence on the structure of the studied glasses, as proved by the trivial effect that the replacement had on T_g_ and NMR-derived Q^n^ distributions of each glass. Nonetheless, it was stated that increasing substitution of Ca with Sr increased the setting times, which was ascribable to the higher basicity of SrO over CaO. Wren et al. (2008) reported 29 and 110 s as the maximum working time and setting time, respectively; which was inadequate for clinical procedures. Sufficient working and setting times were expected to reach 6–10 and 15 min, respectively. However, the optimum biaxial flexural and compressive strength reached 34 and 75 MPa, respectively, proving that these materials could be potentially used in load-bearing applications. The *in vitro* evaluation in SBF showed that all the prepared cements could promote the development of amorphous calcium phosphate at their surface after 1 day of incubation. This event became more apparent (increased density and coverage) over time, representing that these cements could bond to bone directly (Wren et al., 2008).

In line with these researches, Clarkin et al. (2009), Clarkin et al. (2010) employed 4SrO−12CaO−36ZnO−48SiO_2_ (mol%) glass, low molecular weight PAA and a modifying agent, trisodium citrate dihydrate (TSC), to optimize working and setting times which were too short for invasive surgical procedures, including bone fracture fixation and void filling. In their study, the newly-formulated GPC was compared with Hydroset^TM^, a commercial self-setting CPC. They compared compressive strength, flexural strength, Young's modulus, working and setting times, and injectability. The formulation had higher mechanical strength (39 MPa in compression) than both vertebral bone (18.4 MPa) and Hydroset^TM^ (14 MPa). However, the working time (2 min compared to ~4 min for Hydroset^TM^) and rheological properties of the cement, although improved, still required further modifications before their application in minimally invasive surgery, e.g., vertebroplasty or luting applications (Clarkin et al., 2009, 2010).

In their endeavor to adjust the working and setting times of their promising cementitious composites, Wren et al. (2010) added some naturally-derived proteins/polymers to the zinc-containing glass polyalkenoate cements (GPCs). The authors used chitin (Chi.), collagen (Col.), cysteine (Cys.), and keratin (Ker.). They concluded that the addition of these proteins/polymers could lead to little change to the working and setting times, and even the compressive strength was found to decrease slightly. No significant difference was observed in the flexural test. The same GPC containing 4SrO−12CaO−36ZnO−48SiO_2_ (mol%) glass, named Zn-GPC, was compared to commercial materials (Fuji IX and Ketac Molar) which have setting chemistry comparable to Zn-GPCs. Working and setting times (handling properties) for Zn-GPCs were shorter than the commercial materials. Zn-GPCs also had a higher setting exotherm (34°C) than the commercial products (29°C). The maximum compressive strength for Zn-GPC, Ketac Molar and Fuji IX was 75, 216, and 238 MPa, and biaxial flexural strength was 34, 62, and 54 MPa, respectively. Based on the results of compressive strength test, Zn-GPCs have appeared to be more appropriate for spinal applications in comparison to commercial GPCs but the characteristic times for surgical handling still need optimization (Clarkin et al., 2010; Wren et al., 2013).

#### Sr-BGs in Calcium Phosphate Cements (CPCs)

In 2016, Kent et al. (2016) developed a kind of CPCs by reacting BGs with Ca(H_2_PO_4_)_2_ to form cement. They found that a P_2_O_5_ content of 4 mol% or greater is required in SiO_2_−P_2_O_5_−CaO–Na_2_O glass to produce cement. The phases formed depend on glass composition; brushite (CaHPO_4_·2H_2_O) and octacalcium phosphate (Ca_8_H_2_(PO_4_)_6_·5H_2_O, OCP) form first with 6 mol% P_2_O_5_ in the glass. Brushite dissolves, reforms as OCP and then transforms to apatite. These new cements offer a new route to forming CPCs that combine *in situ* setting and injectability of “conventional” CPCs with resorbability and bioactivity of BGs. D'Onofrio et al. (2016) designed and synthesized a series of Sr-doped BGs to add them in a range of CPCs. They aimed to synthesize, as the final product of the cement, Sr-containing HA and investigated the effects of Sr^2+^ ions on the physicochemical properties of the cement. Glasses in the 42SiO_2_−4P_2_O_5_−(39-x)CaO−15Na_2_O–xSrO (mol%) system were synthesized by progressively replacement of Sr with Ca (x = 1.95, 3.90, 9.75, 19.5, 29.25, 39%). Sr-doped CPCs were developed by mixing the glass and Ca(H_2_PO_4_)_2_ powders with a 2.5% solution of Na_2_HPO_4_. Setting time and compressive strength were measured at 1 h, 1 day, 7 and 28 days post-incubation in Tris buffer solution. XRD, FTIR, and radiopacity were measured, too, and crystal morphology was assessed by SEM. Sr substitution in the glass increased setting time up to 25%, while its higher substitutions acted oppositely and resulted in a decrease in the setting time. Compressive strength reached 12.5 MPa because of the interlocking morphology of the crystals. XRD showed that Sr influenced the type of crystal phases formed. Octacalcium phosphate was the main phase present after 1 h and 1 day while after 28 days OCP was completely transformed to Sr-containing HA (Sr_x_Ca(10-x)(PO_4_)_6_(OH)_2_, Sr-HA). Radiopacity enhanced proportionally to Sr substitution in the glass network. This study introduces a novel method regarding the development of a bone graft forming *in vitro* Sr-HA as a final product by applying a Sr-BG as a precursor, and the authors claimed that the prepared injectable cements are promising candidates for orthopedic and dental applications.

#### Sr-doped BGs in Polymeric Cements

Sr-doped BG particles are added in polymeric cements as a bioactive, reinforcing or radiopaque phase. In this regard, Zhang et al. (2015) proposed a new injectable cement composed of Sr-doped borate BG particles (5.5Na_2_O−7.34K_2_O−7.34MgO−20.18CaO−49.54B_2_O_3_−1.83 P_2_O_5_−8.27SrO in mol%) and a chitosan-based bonding phase. The glass was prepared by the conventional melt-quenching route and ground to form particles of <40 μm. The authors prepared the hardening phase via mixing chitosan with a β-glycerophosphate at a ratio of 7:1 by volume. In addition, they prepared the cement paste by mixing the glass particles with the hardening liquid at a ratio of 2.0 g/mL. The BGs stimulated the bioactivity, conversion to HA, and the ability to encourage osteogenesis, whereas the chitosan provided an interconnected biodegradable and biocompatible bonding component. The cement set *in situ* after the initial setting time of 11.6 ± 1.2 min) and represented a compressive strength of 19 ± 1 MPa. The proliferation and differentiation of hBMSCs treated with the cement were significantly higher than the cells incubated with a similar cement made of chitosan-bonded Sr-free borate BG particles. The osteogenic capacity of the cement was shown by micro-computed tomography (micro-CT) and histology of the samples obtained from critical-sized rabbit femoral condyle defects treated with the material. The results showed newly-formed bone at different distances from the implants after 8 weeks. Moreover, the index of bone-implant contact was considerably higher for the implant containing Sr-doped glass compared to the implant with Sr-free glass particles. Overall, the results indicated that this Sr-containing cement could be considered as a promising substitute for the repair and regeneration of irregularly-shaped bone defects with the use of minimally invasive surgery (Zhang et al., 2015).

**Figure 4:**
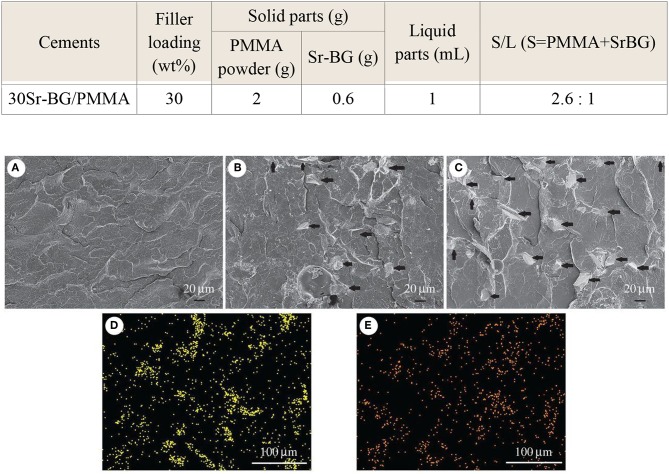
The microstructure of **(A)** PMMA bone cement, **(B)** 10Sr-BG/PMMA and **(C)** 30Sr-BG/PMMA composite cements (the black arrows indicate the Sr-doped BG particles). EDS mapping of the elements **(D)** Si and **(E)** Ca shows that Sr-BG particles are well-dispersed in the 30Sr-BG/PMMA composite cement [Adapted from Cui et al. (2017) with permission from The Royal Society].

Cui et al. (2017) added another Sr-containing borate BG as the reinforcing and bioactive filler to PMMA cement. The PMMA cement and Sr-BG/PMMA composites were prepared by mixing solid and liquid components at particular solid-to-liquid ratios. The solid part of composite cement contained Sr-doped BG (10–50 μm) and PMMA (10–80 μm) particles. The glass composition was 6Na_2_O−8K_2_O−8MgO−16CaO−6SrO−27B_2_O_3_−27SiO_2_−2 P_2_O_5_ (mol%) and prepared by the melting-casting method. The glass and PMMA solid powders were mixed with the liquid component containing 3 mL of MMA monomer and 0.14 μl DMPT accelerator. Detailed compositions of the PMMA cement and Sr-BG/PMMA composite cements are summarized in Table 2 (Cui et al., 2017). Figure 4 shows the microstructure of the PMMA cement and 10Sr-BG/PMMA and 30Sr-BG/PMMA composite cements. The Sr-doped glass powders adhered to the PMMA matrix (Figures 4B,C), and there were some pores within the Sr-BG/PMMA composite cements. Elemental mapping by energy dispersive spectroscopy (EDS) (Figures 4D,E) revealed a homogeneous distribution of silicon and calcium – and hence of glass particles – within the PMMA matrix (Cui et al., 2017).

**Table 2:** Compositions of PMMA cement and related Sr-BG/PMMA cements (Cui et al., 2017).

**Cements**	**Filler loading (wt%)**	**Solid parts (g)**		**Liquid parts (mL)**	**S/L (S = PMMA + SrBG)**
		**PMMA powder (g)**	**Sr-BG (g)**		
Control (PMMA)	0	2	0	1	2: 1
10Sr-BG/PMMA	10	2	0.2	1	2.2: 1
20Sr-BG/PMMA	20	2	0.4	1	2.4: 1
30Sr-BG/PMMA	30	2	0.6	1	2.6: 1

**Figure 5:**
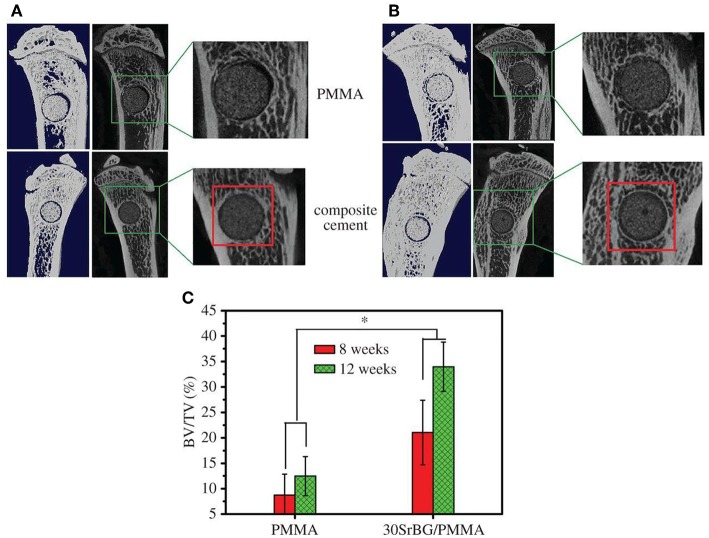
Micro-CT evaluation of bone regeneration in the rat tibia defects after implantation of PMMA and 30Sr-BG/PMMA composite cements. The 3D reconstructed sagittal images of the area surrounding the cement implants show new bone formation around the cement-host bone interface at **(A)** 8 and **(B)** 12 weeks (area outlined in red); **(C)** BV/TV (bone volume/total volume) in the defects implanted with 30SrBG/PMMA composite cement for different post-implantation times. Values are presented as mean ± s.d.; *n* = 3. *Significant difference between groups (*p* < 0.05) [Adapted from Cui et al. (2017) with permission from The Royal Society].

The exothermic polymerization temperature significantly decreased after using Sr-BG/PMMA composite cements compared with BG-free PMMA while the suitable setting time and high mechanical strength were retained. The Sr-BG/PMMA composites were bioactive *in vitro* and released B, Ca, and P ions into SBF. The addition of Sr-doped BG promoted the adhesion, proliferation, migration, and collagen secretion of MC3T3-E1 cells *in vitro*. Moreover, *in vivo* investigation revealed that Sr-BG/PMMA composite cements were better osseointegrated than BG-free PMMA bone cement. Sr-doped BG in the composite cement could enhance new bone formation in rat tibia defects around the cement-bone interface after 8 and 12 weeks post-implantation (Figure 5), while conventional PMMA could only stimulate the formation of an intervening connective tissue layer. As a result, the Sr-BG/PMMA composite cement was recommended as a substitute to PMMA bone cement in clinical orthopedic applications and minimally invasive surgery (Cui et al., 2017).

In order to improve visualization by radiography during surgery, radiopacifying agents such as barium sulfate (Ba_2_SO_4_) or zirconium dioxide (ZrO_2_) are added to cements. These materials may deteriorate the biocompatibility of the cements or have detrimental influences, such as bone resorption, and degradation of mechanical properties (Bhambri and Gilbertson, 1995; Sabokbar et al., 1997; Demian and McDermott, 1998; Wang et al., 2005). However, incorporating bioactive and biocompatibility radiopacifier agents would have beneficial effects. Bioactive radiopaque glasses containing heavy elements such as Sr (Boyd et al., 2009), niobium (Nb) (Bauer et al., 2016), and zirconium (Zr) (Tallia et al., 2014; Montazerian et al., 2015; Montazerian and Zanotto, 2016), known to have positive and therapeutic influences on bone, have attracted the attention of the researchers nowadays. Therefore, O'Brien et al. (2010) incrementally replaced Ba_2_SO_4_ in the commercial Spineplex® cement with a Sr-containing radiopaque glass composition (40SiO_2_−30Na_2_O−20SrO−10CaO in mol%). The substitution increased the setting time from 13.1 min for Spineplex® to 16.6–18.3 min for the new cements. A reduction in the peak exotherm during curing (23°C) was observed for Spineplex® in comparison to the fully replaced cement, demonstrating that reduced thermal necrosis in the *in vivo* setting is achievable using these materials. No significant deterioration was recorded regarding Young's modulus and compressive strength of each formulation due to the addition of Sr-doped BG. Although the radiopacity of the new cements was decreased up to 18% relative to the control, but still maintained radiopacity equal to several millimeters of aluminum (O'Brien et al., 2010).

More recently, Goñi et al. (2018) prepared different composite bone cements comprising PMMA beads and particles of gel-derived SiO_2_-CaO–P_2_O_5_ BGs with 0-20 wt% of CaO substituted with SrO (Mendez et al., 2004). The difference between the cementitious materials was in the Sr content of BG and relative amounts of the solid phase. The aim was to determine the effect of the mixture of solid phase constituents on maximum exothermic temperature, setting time, and injectability. Regarding the obtained results, they stated that composite formulations have improved performance than that of PMMA the reference (PMMA), with lower exotherm temperature and setting time and higher injectability. The same authors showed that incorporation of Sr-substituted BGs into these materials conferred bioactive properties linked to the role of Sr in bone formation, introducing some composite cements that may be appropriate for application in percutaneous vertebroplasty (Goñi et al., 2018).

### Bone Graft Ceramic/Polymeric Composites

Calcium phosphate-based ceramics, e.g., HA and β-tricalcium phosphate (β-TCP), have been extensively using in dental and orthopedic applications since the 1980s (Bohner, 2000), primarily due to their similarity (crystal and chemical properties) to the mineral component of the bone tissue. The supportive role of calcium phosphates regarding adhesion, proliferation, and the differentiation of MSCs and osteoblasts is previously well-documented (Kamitakahara et al., 2008). HA and TCP are both highly osteoconductive and biocompatible. Although HA and TCP are recognized as biocompatible and osteoconductive materials, they suffer from some limitations, such as brittleness and poor mechanical properties. Therefore, a large number of attempts have been made to enhance their mechanical strength, like the addition of a sintering aid to increase the density and minimize the residual porosity of the system. Most of these studies employed BGs and, in particular, 45S5 Bioglass® (Goller et al., 2003) and a CaO-rich BG formulation (2.3K_2_O−2.3Na_2_O−45.6CaO−2.6P_2_O_5_−47.3SiO_2_ in mol%) that is reluctant to crystallization (Bellucci et al., 2014). Additionally, there is proof of the positive effects of the presence of Sr in these materials. To test the influence of the addition of Sr-doped BGs to produce composites, Hesaraki et al. (2012) added different amounts of sol-gel derived Sr-containing BG nano-powders (26CaO−5SrO−5P_2_O_5_−64SiO_2_ wt.%) to HA with a mean particle size of 0.5 μm to improve its mechanical properties after sintering. The samples were sintered at 1,000–1,200°C. A couple of physicochemical and biological assays, including XRD, SEM, microindentation, MTT, and ALP assay were carried out by the authors to characterize the samples. The obtained results showed that the inclusion of 1–10% of BG nano-powder led to the formation of β-TCP phase, the content of which increased with increasing the amount of Sr-BG and temperature. In addition to β-TCP, α-TCP, and calcium phosphate silicate were also found in the composition of HA sintered with 10% glass. After sintering at 1,200°C, bending strength (~70 MPa), microhardness (~300 HV) and fracture toughness (~1.2 MPa.m^1/2^) improved by adding 1–5% Sr-BGs, whereas these mechanical properties decreased when 10% glass was added. The addition of nano-sized Sr-BGs did not alter the rate of cell proliferation but increased the level of ALP produced (Hesaraki et al., 2012). The same gel-derived Sr-BG was added to biphasic calcium phosphate (BCP) by Nezafati et al. (2014), who sintered the mixture at 1,100, 1,200, and 1,300°C. The maximum bending strength (45 MPa) was achieved when BCP was added with 3 wt% Sr-BGs and sintered at 1,200°C. The addition of Sr-BGs did not affect the phase composition of BCP when it was treated at 1,200°C, and the composite supported the adhesion and expansion of rat calvarium-derived osteoblasts.

Sr-containing phosphate-based glass (45P_2_O_5_−32SrO−23Na_2_O in wt%) was also used as a sintering aid for β-TCP, which was heat-treated at 1,250°C (He and Tian, 2018). The results showed that the glass addition allowed liquid-phase sintering of β-TCP with the noticeable promotion of densification (He and Tian, 2018). In the sintering process, the Sr-doped BG reacted with β-TCP, and the Sr^+2^ ions replaced Ca^2+^ ions of β-TCP. Furthermore, the glass addition efficiently hindered the transformation of β-TCP to α-TCP. The compressive strength of these porous β-TCP-based bioceramics was improved from 7 to 11 MPa by introducing 10 wt% Sr-doped BGs.

It has also been proved in several other studies that Sr-substituted TCP and HA cements/ceramics show promise for use in orthopedic, e.g., in filling bone defects (Kim et al., 2004; Saint-Jean et al., 2005; Pina et al., 2010). More recently, Kuda et al. (2018) have prepared composite materials by adding 1 wt% SrO to biogenic HA (BHA) and sodium borosilicate glass in a ratio of 50/50 by weight. The composites were sintered at 780°C for 1 h. The BG addition improved the sinterability, while the crystal lattice constant of biogenic HA decreased. It was also found that such BHA/glass/SrO composite possessed a higher porosity and rate of dissolution in a physiologic solution, which make it highly attractive for use in the replacement of defective areas of bone (Kuda et al., 2018).

**Figure 6:**
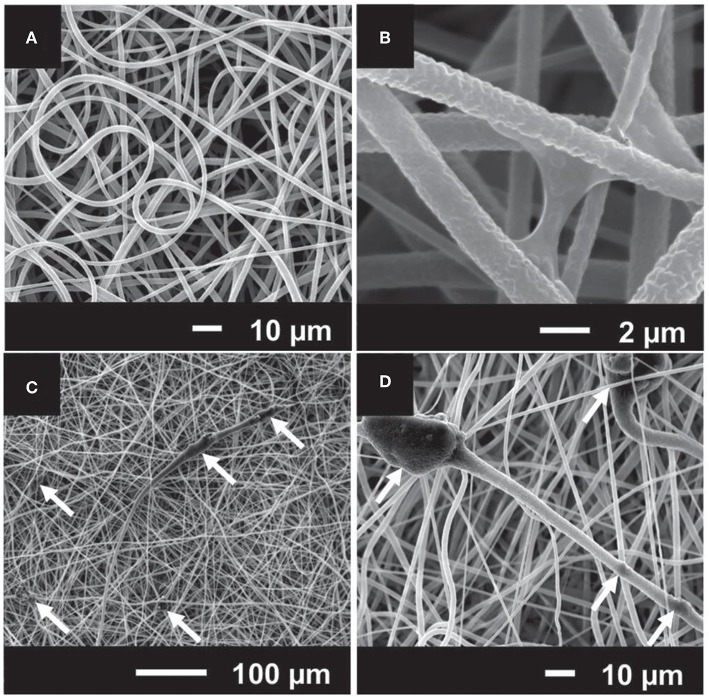
SEM micrographs of electrospun composites made of **(A)** and **(B)** poly(caprolactone) (PCL); **(C)** and **(D)** PCL and particles of Sr-containing BG. The white arrows indicate that BG particles are embedded in the polymeric electrospun fibers [Adapted from Santocildes-Romero et al. (2016), after permission by Wiley and Sons].

Other promising applications of Sr-doped BGs are addressed to the repair of alveolar bone in dentistry. Polymeric membranes are one of the extensively used materials in periodontology and dental implantology, which improve the bone healing process in the method of guided bone regeneration (GBR) (Misra et al., 2006). The standard rule of this treatment is to physically provide a relatively remote environment where bone can repair via keeping out local soft tissues from a defect site. An ideal barrier membrane should resorb and stimulate bone tissue regeneration within the defect (Misra et al., 2006). The prospective of electrospinning (Agarwal et al., 2008) and advantages of Sr-substituted BGs have been discussed for this purpose, and it is expected that the combination of these two approaches results in the fabrication of potent membranes in terms of bone tissue regeneration. Ren et al. (2014) combined melt-electrospun polycaprolactone (PCL) with Sr-containing 45S5 BG to prepare composite scaffolds. They did not include 45S5 BG as reference material, so it was uncertain whether the final device benefitted from the substitution of Ca with Sr. Furthermore, Ren et al. (2014) reported the diameter of the fibers in the range of several tens of micrometers as a result of electrospinning of melt. It has been stated that fibers with smaller diameters (a few micrometers to hundreds of nanometers) are more favored for bone tissue engineering strategies due to their close similarity in size with bone tissue's collagen fibrils (Holzwarth and Ma, 2011); this outcome may be achieved through solution-electrospinning. Therefore, Santocildes-Romero et al. (2016) developed composite electrospun membranes made of a bioresorbable PCL and particles of Sr-substituted BG, which demonstrated osteogenic potential (Santocildes-Romero et al., 2015), and assessed their potential for bone tissue regeneration. The electrospun fibers exhibited porous surfaces and some regions of increased diameter where the particles were accumulated; interestingly, the Sr-doped BG particles were observed both inside and on the surface of the fibers (Figure 6). The glass dissolved after immersion in water, releasing alkaline ions that are related to increased pH. Further evidence suggested that pH changes is controlled or even reduced due to the accelerated polymer degradation, which offsets the pH variation after glass dissolution. All compositions were biocompatible *in vitro* after being tested with rat osteosarcoma cells, except for the membranes with more than 50 μg of glass on their surface (Santocildes-Romero et al., 2016).

In another novel research, Fernandes et al. (2016) fabricated a composite membrane by combining poly-L-lactic acid (PLLA) with 10 wt% Sr-doped borosilicate BG (0.05Na_2_O–xMgO–yCaO– (0.35-x-y)SrO−0.20B_2_O_3_−0.40SiO_2_ in molar ratio, where x, y = 0.35 or 0.00, and x ≠ y) using electrospinning. Smooth and uniform fibers (1–3 μm in width) with a homogeneous distribution of Sr-doped BG microparticles (sizes < 45 μm) were obtained. Degradation studies, performed in phosphate buffered saline, revealed that the inclusion of Sr-doped BG particles into the PLLA membranes accelerated the degradability and enhanced the water uptake; furthermore, a continuous release of cations from the glass was observed. The addition of glass particles increased the mechanical properties of the membranes: specifically, Young's modulus and tensile strength increased by about 69 and 36 %, respectively. Additionally, cellular *in vitro* evaluation confirmed that the membranes enhanced the osteogenic differentiation of BMSCs as verified by increased ALP activity and up-regulated osteogenic gene expression (Alpl, Sp7, and Bglap) in comparison to PLLA alone. This study further suggests that such composites have great potential as effective biomaterials capable of promoting bone regeneration (Fernandes et al., 2016).

### Coatings

One approach for solving the mechanical restrictions of BGs for load-bearing applications is to apply them as a coating on a mechanically strong and tough substrate. BG coatings for biomedical applications were the subject of many studies and have been comprehensively reviewed by Rawlings (1993), Niinomi (2010), Cao and Hench (1996), Verné (2012), Xuereb et al. (2015), Marghussian (2015), and Montazerian and Zanotto (2016). Many promising BGs including Sr-doped BGs have been studied to coat Ti, ZrO_2_, Al_2_O_3_, stainless steel, and glass-ceramic implants. Many methods, such as enameling, sputtering, flame spraying, laser deposition, plasma spraying, and electrophoretic deposition (EPD), have been attempted for application of “perfect” coatings (Verné, 2012). Sr-containing glasses have also been utilized to develop coatings over implants. For example, Lotfibakhshaiesh et al. (2010) were interested in determining how SrO substitution for CaO (0, 10, 25, 50, 75, and 100%) affects sintering and crystallization of 49.96SiO_2_−7.25MgO−3.30Na_2_O−3.30K_2_O−3.00ZnO−1.07 P_2_O_5_−32.62CaO (mol%) glass (Gentleman et al., 2010) coating. Amorphous coatings on Ti-6Al-4V alloy produced by enameling showed good adhesion to the substrate except for the 100% Sr-substituted coating. Substituting Sr for Ca reduced the glass transition temperature and increased the onset temperature of crystallization. The mixed Ca/Sr glasses exhibited a larger sintering range (i.e., the temperature range between glass transition and crystallization), which favors glass processing without crystallization and obtaining amorphous well-sintered coatings. On the other hand, complete substitution led to crystallization and reduced the temperature range for sintering (Lotfibakhshaiesh et al., 2010). One of these resistant-to-crystallization glasses, having thermal expansion coefficient (TEC) similar to HA, was employed by Newman et al. (2014) for *in vivo* investigations, too. The coating was applied to roughened Ti-6Al-4V, and it produced no unfavorable tissue reaction following implantation into the distal femur and proximal tibia of twenty-seven New Zealand White rabbits for 6, 12, or 24 weeks. In this research, the glass dissolved over 6 weeks, stimulating enhanced peri-implant bone formation compared with HA-coated implants in the contralateral limb used as controls. Moreover, superior mechanical fixation was reported in the Sr-doped BG group after 24 weeks of implantation (Newman et al., 2014).

Miola et al. (2015) modified the original 45S5 BG composition by introducing 6 mol% of ZnO and/or SrO in place of CaO. SEM and XRD analyses proved that Zr and Sr addition did not significantly modify the glass structure while EDS analysis verified the presence of these elements in the glass composition. Sr-containing glasses were mixed with chitosan to synthesize organic-inorganic composite coatings on stainless steel (AISI 316L) by EPD. Tape and bending tests revealed a good coating-substrate adhesion for coatings made from Sr-doped 45S5 and Zn/Sr-codoped 45S5 glasses, whereas the adherence to the substrate decreased by using Zn-doped 45S5 glass. Microstructural analyses showed the composite character of coatings and indicated that the glass particles were well-embedded into the polymeric matrix, and the coatings were relatively uniform and crack-free. Although the bioactive behavior of the Sr-containing coating was confirmed after immersion in SBF, the coatings containing Zn exhibited no bioactivity.

**Figure 7:**
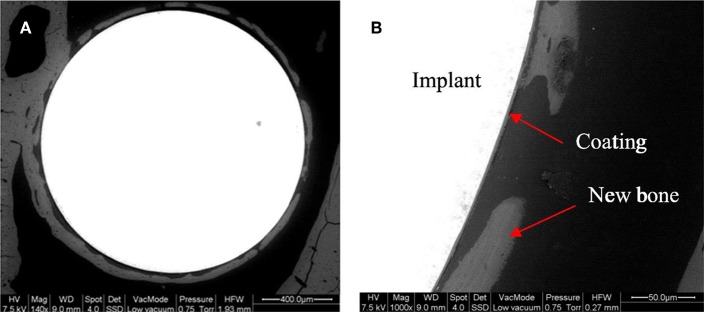
Backscattering SEM images of a Sr-doped 45S5 glass coating after 4 weeks of implantation in the rat femur **(A)**. A detail of the different portions of the system is shown with arrows **(B)** [Adapted from Omar et al. (2015), after permission by Elsevier].

Later, Omar et al. (2015) utilized another organic-inorganic composite coating to protect the stainless-steel implant and generate a barrier for metallic ion dissolution by using the potential of BG particles. The composite was made by a sol-gel method containing TEOS, methyl-triethoxysilane (MTES), colloidal silica nanoparticles, and 45S5 BG particles in which 2 mol% of CaO was substituted with SrO. The corrosion resistance and bioactivity of the stainless steel coated with this composite were evaluated *in vitro* and *in vivo* to analyze bone formation. The coating system provided outstanding protection against aggressive fluids. The formation of HA was observed after 30 days of immersion in SBF. *In vivo* tests in Wistar–Hokkaido rat femur after 4 or 8 weeks showed minor differences in the thickness of newly formed bone observed by SEM and noteworthy changes in bone quality. The *in vivo* reaction of the coatings containing Sr-doped BG was successful in the early stages of implantation regarding the bone morphology and quality (Figure 7) (Omar et al., 2015).

More recently, Molino et al. (2017) developed a hierarchical scaffold with a trabecular architecture mimicking that of cancellous bone via EPD of Sr-doped mesoporous BG (MBG) particles on the surface of sponge replicated strong but inert glass-ceramic scaffold. The spherical particles of mesoporous SiO_2_−CaO–SrO glass was synthesized by aerosol-assisted spray drying that, after being deposited on the glass-ceramic scaffold walls, stimulated a fast apatite-forming ability. Such a meso-macroporous hierarchical implant was suggested as a bioactive and mechanically strong implant for potential applications in bone tissue engineering.

### Glass-Ceramics

It is known that bioactive GCs are designed for at least two main objectives: (i) improving the mechanical properties of glasses or (ii) benefiting from a particular characteristic of crystals (Montazerian and Zanotto, 2016). For example, needle-like apatite in many GCs improves mechanical properties, resembles the biological apatite in bone, and enhances the aesthetic features of dental GCs (Montazerian and Zanotto, 2017b). In this regard, the conversion of Sr-containing glasses into glass-ceramic in which different type of crystals, e.g., Sr-doped phases, are crystallized would have some beneficial effects. Therefore, (Topalović et al., 2017) have developed glass-ceramics in the 42P_2_O_5_−40CaO−5SrO−10Na_2_O−3TiO_2_ (mol%) system through the sintering of melt-quenched glassy frits. They neither detected a phase containing Sr nor measured mechanical properties. However, they observed the formation of HA on the glass-ceramic surface immersion of the GCs for 21 days in SBF solution (Topalović et al., 2017). Dessou *et al*. have also reported no adverse influence on the bioactivity mechanism after increasing Sr content which leads to the formation of strontium silicate and Sr-doped diopside (MgCaSi_2_O_6_) in the thermally treated gel-derived BG powder discussed in section Sr-containing BGs: an overview (Dessou et al., 2017). We agree with them that the new glass-ceramic can be employed for the synthesis of Sr-containing/releasing scaffolds for bone tissue repair or engineering. Nevertheless, to examine the kinetics of Sr release and to provide insights into its beneficial effect on cell attachment, proliferation differentiation, and also mechanical properties, further work should be conducted.

Additionally, Kapoor et al. (2013) have studied the influence of SrO and ZnO co-dopants on thermo-mechanical behavior of alkali-free bioactive glass-ceramics of (36.07-x)CaO–xSrO– (19.24-y)MgO–y ZnO−5.61P_2_O_5_−38.49SiO_2_−0.59CaF_2_ (x = 2–10, y = 2–10) (mol%). They could sinter the glass powders before the onset of crystallization, leading to well-sintered and mechanically strong glasses/glass-ceramics after heat treating for 1 h at 800, 850, and 900°C. The crystalline phases of diopside and fluorapatite [Ca_5_(PO_4_)_3_F] with partial replacement of Ca for Sr with increasing strontium contents were detected in the densified specimens (Kapoor et al., 2013). The substitution of SrO with CaO led to the partial replacement of Ca^2+^ by Sr^2+^ in the fluorapatite and diopside crystal structures. The maximum flexural strength of ~150 MPa and Weibull modulus of ~19 were recorded for the samples sintered at 850°C. In another research, Cai et al. (2011) have sintered Sr and Mg co-doped calcium phosphate gel-glasses to prepare GCs. XRD data revealed the presence of Ca_4_P_6_O_19_ and β-Ca_2_P_2_O_7_ for all heat-treated samples, and amorphous glass decreased with the increase of the heat treatment temperature. A fast release of Mg^2+^ ions from the residual glass was documented in the case of the glass-ceramics obtained by heat treatment at 700°C. This event supports the formation of amorphous apatite deposition. Although, the presence of Mg^2+^ ions in the solution resulted in a delay in the crystallization of apatite layer, and induced dissolution/precipitation dynamic processes on the glass-ceramic surface and an unstable surface that had a detrimental effect on cell attachment and proliferation during *in vitro* cell culture procedure. On the contrary, less glassy phase, and a stable apatite layer were observed in the samples heat-treated at 760 and 780°C, resulting in creating a proper surface for cell growth and differentiation. Sr element, preliminary existed in the glassy phase, was later detected in all the deposited apatite particles on sample surface immersed in SBF, indicating that the incorporated Sr was capable of substituting into the apatite nuclei and favoring its crystallization. It seems that the release of Sr^2+^ ions from glassy phase or Sr-doped crystals, e.g., Ca_4_P_6_O_19_, in GCs offer an insight into the choice of a sintered Sr-containing GCs for the development of three-dimensional porous scaffolds for bone tissue engineering. However, further focus on the effect of these crystals on the biological and mechanical properties of GCs are required. Table 3 summarizes different characteristics of Sr-doped or Sr-BGs containing materials described in section Bone cements.

**Table 3:** Physical, chemical, and biological properties of some Sr-doped or Sr-BGs containing materials described in section Bone cements.

**Material type and composition**	Physical properties^*^	Mechanical properties^*^	***In vitro / In vivo* properties**	**References**
Sr-doped hydroxyapatite (HA)	Final setting time: 15–18 min Setting temperature: max. 58°C	Compressive strength: ~41 MPa Bending strength: ~31 MPa Elasticity modulus: ~1.5 GPa	Improving the osteoblast adhesion and mineralization *in vitro* and bone growth and osseointegration *in vivo*	Cheung et al., 2005; Ni et al., 2006
Sr-doped calcium phosphate-based cement (CPC)	Initial and final setting of 8–10 min and ~15 min, respectively	Compressive strength: ~12 MPa	Promoting cell proliferation and ALP activity of MG-63 cells cultured on the cement doped with Sr	Kuang et al., 2012
Series of ionomer cements containing SiO_2_-ZnO–CaO–SrO based BGs	Working and setting time still have to be adjusted	Compressive strength: 39–75 MPa Flexural strength: 34–62 MPa	Have to be evaluated after optimizing physical and mechanical properties	Boyd et al., 2008; Wren et al., 2008, 2010, 2013; Clarkin et al., 2009
CPC containing 42SiO_2_-4P_2_O_5_-(39-x)CaO−15Na_2_O–xSrO (in mol%, x = 1.95, 3.90, 9.75, 19.5, 29.25, 39)	Final setting time: 20-40 min	Max. ompressive strength: 12.5 MPa	Forming *in vitro* Sr-doped HA	D'Onofrio et al., 2016
Chitosan-based cement containing 5.5Na_2_O−7.34K_2_O−7.34MgO−20.18CaO−49.54B_2_O_3_−1.83P_2_O_5_−8.27SrO in mol% borate BGs	Initial setting time: 12 min	Max. compressive strength of 19 MPa	Enhancing the proliferation and osteogenic differentiation of hBMSCs *in vitro* New bone in rat tibia after 8 weeks	Zhang et al., 2015
PMMA-based cement containing 6Na_2_O−8K_2_O−8MgO−16CaO−6SrO−27B_2_O_3_−27SiO_2_−2P_2_O_5_ (mol%) in mol% borosilicate BGs	Final setting time: 8–12 min	Max. compressive strength of 78–88 MPa Flexural strength: 50–60 MPa Elasticity modulus: ~2.5–2.7 GPa	Promoting the adhesion, migration, proliferation, and collagen secretion of MC3T3-E1 cells *in vitro* New bone formation in rat tibia after 8–12 weeks	Cui et al., 2017
PMMA-based cement (Spineple^®^) containing 40SiO_2_-30Na_2_O−20SrO−10 CaO in mol% BGs	Setting time: 16–18 min Setting temperature: 50–75°C	Max. compressive strength of 75–100 MPa Elasticity modulus: ~2.5–2.7 GPa	–	O'Brien et al., 2010
PMMA-based cement (Spineple^®^) containing SiO_2_-CaO–P_2_O_5_-SrO gel-derived BGs	Setting time: 16–20 min Setting temperature: ~34–45°C	Max. compressive strength: ~100 MPa Elasticity modulus: ~1.5–2.5 GPa	–	Goñi et al., 2018

* Minimum requirement set by ISO 5833 for polymeric cements:

Max. peak temperature:: 908°C.

Setting times for operation in a surgical room 6–15 min.

Compressive strength > 70 MPa.

Flexural strength > 50 MPa.

*Elasticity modulus > 1.8 GPa*.

## Three-Dimensional (3D) Scaffolds

Sr-doped BGs have also been processed to acquire a porous structure at different size scales (from macro- to meso-range), thus obtaining tissue engineering 3D scaffolds allowing bone ingrowth and regeneration.

Sr-containing BG scaffolds were first prepared by two independent research groups from China Zhu et al. (2011) and Japan Wang et al. (2011) in 2011 by using MBGs as starting materials. Specifically, CaO-SrO-SiO_2_-P_2_O_5_ MBG scaffolds were fabricated by the combination of polyurethane foam and block copolymer EO_20_PO_70_EO_20_ (P123) as co-templates and evaporation-induced self-assembly (EISA) process, according to a strategy pioneered in 2007 by Li et al. (2007) for the production of macro-mesoporous hierarchical scaffolds. These Sr-doped MBG scaffolds exhibited an interconnected macroporous 3D network with a pore diameter in the range of 100 to 400 μm, which closely mimicked the trabecular architecture of cancellous bone and mesoporous walls with mesopore size of 4–5 nm. They showed no significant difference in terms of phase composition (being amorphous), macroporous structure, and textural characteristics compared to the Sr-free scaffolds used as a reference. The release of Sr^2+^ ions into the culture medium was reported to enhance the proliferation and ALP activity of mesenchymal stem cells (MSCs)(Wang et al., 2011) as well as the osteogenic expression of MC3T3-E1 osteoblast-like cells (Li et al., 2007).

Interestingly, the osteogenesis/cementogenesis-related gene expression of periodontal ligaments cells (harvested from human patients) was also observed to be stimulated by increasing concentrations of Sr released by MBG foams, thus suggesting the suitability of these materials for periodontal tissue engineering applications (Wu et al., 2012). This early *in vitro* evidence was further corroborated by the results from a study in an osteoporotic animal model of bilaterally ovariectomized rats (Zhang et al., 2014a). Specifically, Sr-doped MBG scaffolds were reported to significantly increase alveolar bone regeneration in the periodontium of osteoporotic rats at 1 month after surgery (bone formation: 46.67%) as compared to the Sr-free MBG group (39.33%) and unfilled controls (17.50%). Furthermore, osteoclasts were significantly reduced in defects receiving Sr-MBG scaffolds, as expected from the ability of Sr to halt bone resorption. Analogous results about the ability of Sr-doped MBG scaffolds to promote bone formation and reduce osteoclast activity were reported after implanting the graft in critical-size defects created in the femur of ovariectomized rats (osteopenic animal model) (Zhang et al., 2013; Wei et al., 2014).

**Figure 8:**
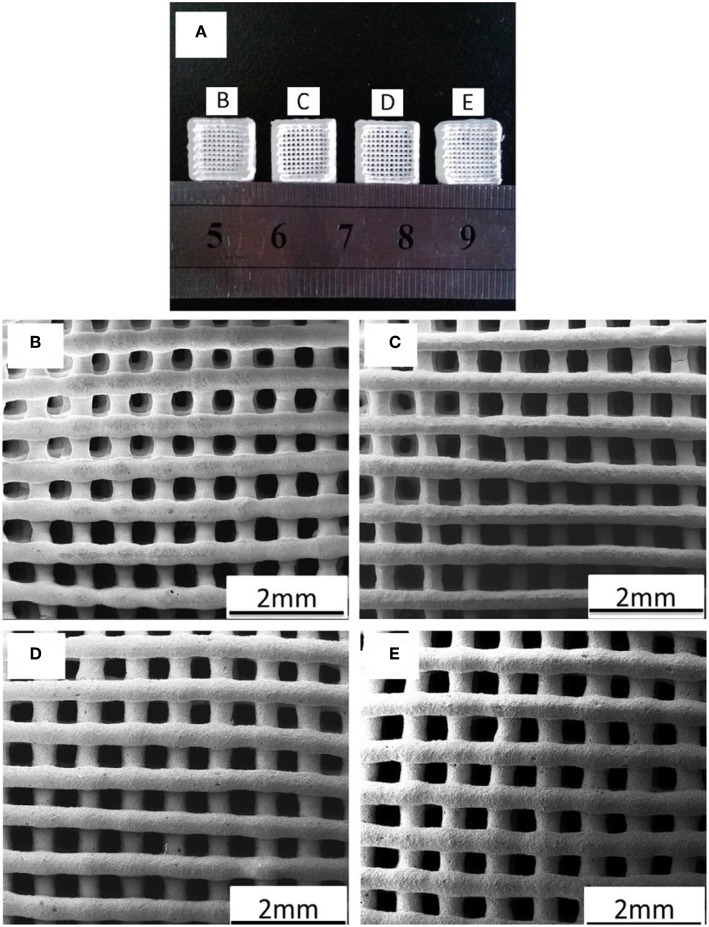
3D printing of Sr-doped MBGs: **(A)** photograph of 3D-printed scaffolds; SEM images of the MBG scaffolds doped with **(B)** 0 (control), **(C)** 5, **(D)** 10, and **(E)** 20 mol% of Sr. Images adapted from Zhang et al. (2014b).

Despite the attractive textural and biological properties, MBG foams produced by co-templating (polymeric sponge + EISA) often suffer from a major limitation, namely high brittleness (compressive strength about 50–250 kPa (Wu et al., 2010, 2011, 2013), which make them difficult to easily handle by surgeons and to easily apply in clinics. Therefore, other processing routes have been experimented to increase the mechanical properties of MBG scaffolds to more acceptable levels for bone repair applications. Zhang et al. (2014b) first reported the use of 3D printing to fabricate hierarchical Sr-doped MBG scaffolds by extruding and depositing filaments of glass powders bound together with poly(vinyl alcohol) gel according to a layer-wise approach (Figure 8). These scaffolds, retaining the mesoporous texture in the glass walls along with a grid-like macroporous structure, exhibited a compressive strength (8–9 MPa) that was about 170 times higher than that of sponge-templated MBGs, and an excellent apatite-forming ability in SBF. Furthermore, the fabricated samples could promote the proliferation and differentiation of osteoblastic cells *in vitro* and showed attractive properties for sustained drug release (model drug: dexamethasone) for use in local drug delivery therapy, due to their inherent mesoporous structure. After being implanted in critical-size calvarial defects of rats, these 3D-printed Sr-doped MBG scaffolds revealed superior osteoinductive properties and pro-angiogenic ability to enhance bone formation compared to Sr-free MBG controls (Zhao et al., 2015). On the other hand, major general drawbacks of 3D-printed MBG scaffolds compared to sponge-replicated ones include higher manufacturing cost and loss of the typical bone-like trabecular architecture, which is replaced by a more simplified grid-like array of micro-sized channels (Baino et al., 2016).

Instead of using sol-gel MBGs, Erol et al. (2012) fabricated macroporous SiO_2_-CaO-P_2_O_5_-SrO glass scaffolds by sponge replication starting from melt-derived glass powder. These scaffolds were bioactive, as demonstrated by the formation of a surface HA layer after immersion in SBF, and able to act as vehicles for the local release of therapeutic Sr^2+^ ions. However, the kinetics of both apatite formation and Sr delivery were significantly lower and less finely controllable compared to Sr-doped MBGs, due to the absence of a mesoporous texture—and hence a lower specific surface area (Izquierdo-Barba and Vallet-Regí, 2015)—in melt-quenched materials. Furthermore, the compressive strength of these Sr-containing BG foams (0.1 MPa) was one order of magnitude lower than that of cancellous bone; some improvements (1.4 MPa) were obtained by the coating of the scaffold struts with gelatin, which performed a crack-bridging action. Özarslan and Yücel, 2016) produced Sr-doped glass scaffolds by the same method using rice hull ash as a sustainable source for SiO_2_ and obtained analogous results to Erol et al. (2012).

Given its importance in the context of bone tissue engineering scaffolds, the bioactivity mechanism of Sr-containing melt-derived BGs was studied by Sriranganathan et al. in detail (Sriranganathan et al., 2016). It was observed that Ca replacement with Sr in the glass composition could retard the formation of a HA-like phase. It was proposed that the formation of apatite happens through an octa-calcium phosphate precursor phase, which subsequently transforms to HA. However, the equivalent octa-Sr phosphate does not exist, and hence, in the absence of Ca, apatite formation does not occur.

A few studies have recently dealt with the incorporation of Sr-doped BG particles in polymeric matrices to obtain composite scaffolds, such as electrospun polycaprolactone fibrous meshes (Ren et al., 2014), gelatin-based freeze-dried bone grafts (Jalise et al., 2018; Zhao et al., 2018), and acrylic gel (Zhang et al., 2017). In general, all these studies have emphasized the role of Sr-containing glass inclusions to impart osteogenic and angiogenic properties to the non-bioactive polymeric matrix. Early extrusion tests were also performed to produce composite scaffolds by 3D printing of type I collagen gel enriched with spray-dried SiO_2_-CaO-SrO MBG submicronic spheres (Montalbano et al., 2018).

In a very interesting study, Zhang et al. (2017) fabricated 3D-printed polycaprolactone/Sr-doped MBG composite scaffolds and tackled the ambitious challenge of finding a correlation between macroporous characteristics of the implant and biological response of bone cells. For this purpose, three batches of composite scaffolds were prepared in which the angles between the latitudes and longitudes of printed filaments were set to 45, 60 and 90°, and then the proliferation and ALP activity of MC3T3-E1 cells were tested to assess any difference due to macropore geometry. It was shown that the cell proliferation rate on the 45°-oriented scaffolds was slightly higher than that on the other types after 1 and 4 days, but there was a significant increase at 1 week. Furthermore, the 45°-oriented scaffolds were associated with a significant increase in ALP activity of cells compared to the other groups after 2 weeks. These early results, suggesting that an orientation of 45° among the scaffold struts could be more favorable to promote cell proliferation and osteogenic differentiation, represent an important step toward the development of optimal scaffold design and motivate further investigation on this topic.

**Figure 9:**
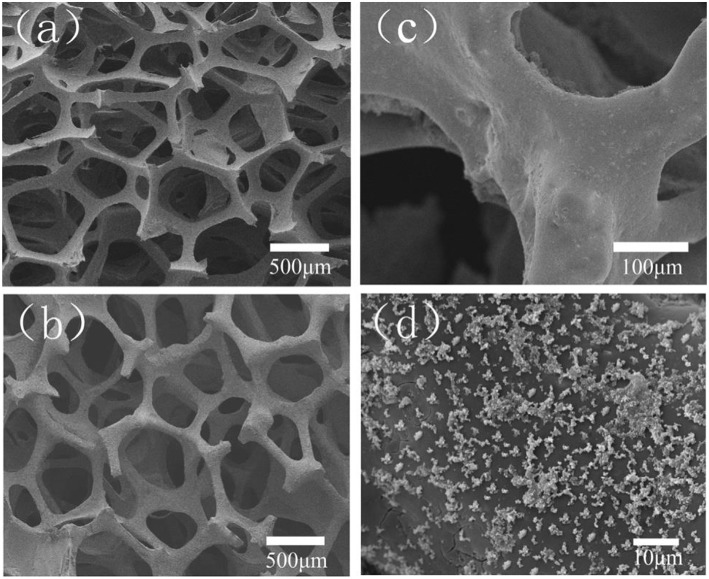
Borosilicate 13-93B2 glass foam doped with 6 mol% of Sr: **(a)** polyurethane foam used as a template; **(b,c)** foam-replicated scaffold; **(d)** apatite agglomerates formed on the surface of the scaffold after immersion in SBF for 5 days. Images adapted from Yin et al. (2018) with permission.

Most of the available studies focus on Sr-doped silicate glasses, produced by either melting or sol-gel-like strategies (MBGs); however, Yin et al. (2018) recently reported the fabrication of sponge-templated scaffolds using melt-derived borosilicate glasses with high B_2_O_3_ content (13-93B2 basic composition (18SiO_2_–36B_2_O_3_–22CaO–6Na_2_O–8K_2_O–8MgO–2P_2_O_5_ mol%) doped with 3, 6, and 9 mol% SrO). Borosilicate glasses are attractive for tissue engineering applications being characterized by higher reactivity with biological fluids—and hence faster apatite-forming kinetics—compared to silicate glasses, but high amounts of boron ions can be toxic to cells (Balasubramanian et al., 2018). It was reported that Sr-doped 13-93B2 glass scaffolds were bioactive and had suitable features for bone repair (bone-like trabecular structure, porosity 80 vol.%, pore size 200–500 μm, compressive strength 11 MPa) (Figure 9); moreover, very interestingly, the increase of Sr concentration in the glass formulation accelerated the release of silicate and Ca^2+^ ions, known for having a stimulatory effect on osteogenesis (Hench, 2009) and angiogenesis (Kargozar et al., 2018d), and significantly reduced the release of boron ions in some extent. Therefore, doping with particular proportions of SrO was suggested as a mean to decrease or even suppress the rapid release of boron, thus minimizing its cytotoxicity.

## Biological Functions of Strontium

### Osteogenesis Induction

The main concept behind adding Sr to BG structure is related to its potential of improving the proliferation and the differentiation of pre-osteoblastic cells into osteoblasts via the activation of various cell signaling pathways such as Ras-mitogen-activated protein kinase (Ras/MAPK) (Peng et al., 2009). Sr can bond to the calcium-sensing receptor (CaRS) and, thereby, activates Ras/MAPK resulting in the up-regulation of genes involved in osteogenesis (e.g., Runx2) (Schindeler and Little, 2006). The activation of the NFATc1 signaling pathway is another mechanism by which Sr^2+^ ions can promote osteogenesis. This signaling pathway can up-regulate Wnt3A mRNA expression and trigger β-catenin dependent Wnt/β-catenin signaling pathway in MSCs (Yang et al., 2011). It has been previously shown that Wnt/β-catenin signaling plays an important role in bone development and homeostasis as well as regulation of the commitment of the differentiation of stem cells into osteoblastic lineages during fracture healing (Chen et al., 2007). Furthermore, Sr may activate the osteogenic differentiation of MSCs through the production of cyclo-oxygenase 2 (COX-2)-mediated prostaglandin E2 (PGE2) (Marie, 2007). Regarding the mentioned data, several researchers worldwide investigated the potential of Sr-containing glasses to accelerate bone repair and regeneration. As an illustration, Santocildes-Romero et al. prepared a series of 45S5 BGs in which Ca was partially (50%) or fully (100%) substituted by Sr (named as Sr50 and Sr100, respectively) and evaluated the osteogenic effects of the materials produced (Santocildes-Romero et al., 2015). The results of real-time PCR showed that the incubation of MSCs with 13.3 mg/mL of Sr50 and ≥6.7 mg/mL of Sr100 actually resulted in up-regulation of some specific bone-related genes like Alpl and OCN. In another study, Lao et al. using particle-induced X-ray emission (PIXE) technique showed that biomineralization in the defect sites of the distal epiphysis of the femur of adult male New Zealand White rabbit implanted with 5 wt.% Sr-containing sol-gel BGs is significantly higher than Sr-free glasses (Lao et al., 2013). The authors concluded that this event could be correlated with the delivery of Sr^2+^ ions up to several ten microns around the implanted Sr-doped glass particles.

It is noteworthy that the osteogenic effect of Sr is dose-dependent (Verberckmoes et al., 2003). It has been shown that Sr at low concentrations (25–500 μM) promotes the osteogenic differentiation of cells, while it has an inverse effect on the differentiation at high concentrations (1,000–3,000 μM). In fact, Sr at higher doses induces apoptosis via the phosphorylation of ERK1/2 signaling molecules followed by the downregulation of Bcl-2 and increasing the phosphorylation of BAX (Aimaiti et al., 2017).

### Osteoclastogenesis Inhibition

**Figure 10:**
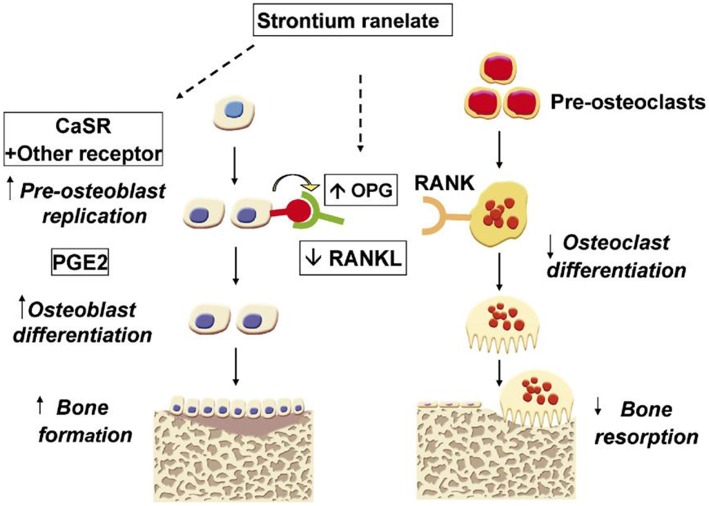
The mechanisms proposed for the action of strontium ranelate (SrR) on the bone cells. SrR can stimulate bone formation through activating some well-known receptors such as the calcium-sensing receptor (CaSR) and increasing prostaglandin E2 (PE2) production by osteoblasts. In contrast, SrR can inhibit bone resorption via increasing osteoprotegerin and decreasing receptor activator of nuclear factor kappa B ligand (RANK) expression by osteoblasts. Adapted from Marie (2007) with permission.

In addition to osteoblastogenesis, Sr plays a significant role in the osteoclastogenesis (see Figure 10). In fact, Sr is known to reduce osteoclast differentiation, activity, and bone resorption (Baron and Tsouderos, 2002).

Receptor activator of nuclear factor kappa-B ligand (RANKL) is a molecule with the ability to affect bone regeneration and remodeling. This molecule can bind to RANK on cells of the myeloid lineages (e.g., osteoclast precursor cells) and act as a major factor for improving the differentiation and activation of osteoclasts (Boyle et al., 2003). Strontium causes a reduced pre-osteoclast differentiation into osteoclasts via down-regulating the RANKL expression and also up-regulating its antagonist, i.e., osteoprotegerin (OPG), Saidak and Marie (2012). Moreover, it has been documented that Sr (as SrR formulation) at high concentrations can stimulate apoptosis in osteoclasts through the PKC βII pathway (Hurtel-Lemaire et al., 2009). With respect to this evidence, (Gentleman et al., 2010) evaluated the effects of Sr-substituted BGs on osteoblasts and osteoclasts *in vitro* (Gentleman et al., 2010). They developed a series of BGs based on SiO_2_−P_2_O_5_−Na2O–CaO formulation in which 0–100% of the CaO was substituted by SrO. Their results revealed that the release of Sr^2+^ ions from the glasses into cell culture medium enhances metabolic activity in osteoblasts, whereas the activity of osteoclasts inhibits (RAW264.7 monocytes). The last event is approved through the reduction of tartrate-resistant acid phosphatase activity and the inhibition of resorption of calcium phosphate films in a dose-dependent manner. The authors stated that the inhibition of osteoclast differentiation and disruption of cytoskeletal elements are the two main reasons by which the Sr-substituted glasses act.

### Antibacterial Properties

The antibacterial effect of Sr has not been well-understood and is an open issue of research. Some researchers have reported that Sr has an inhibitory effect on various strains such as *Escherichia Coli* (E. coli) and *Porphyromonas gingivalis* (P. gingivalis) (Zhang et al., 2014c; Liu et al., 2016). For instance, Brauer et al. showed that the bactericidal action of injectable bone cements based on BGs could be increased via Sr substitution (Brauer et al., 2012). They reported that the samples containing small amounts of Sr (2.5 mol%) reduce the number of bacteria *(Streptococcus faecalis*) up to one order of magnitude as compared to Sr-free samples. However, the concentrations above 0.16 mmol/L (14 ppm) exhibited no further bactericidal action. One of the mechanisms by which Sr elicits its antibacterial activity may be attributed to its potential to depress intracellular polysaccharide (IPS) accumulation in bacteria such as *Streptococcus mutans* (Wegman et al., 1984). It has been shown that IPS plays an important role in the persistence of bacteria in excess sugar environments (Busuioc et al., 2009). Another proposed mechanism for the antibacterial effect of Sr is related to its intervention to bacterial metabolism at concentrations above 180 mM since Sr^2+^ ions can act as a competitor to iron for specific binding sites in iron-sensing proteins (Brown et al., 2006). In contrast to the studies mentioned above, other scientists showed that the release of increased levels of Sr^2+^ has no positive effect on the bacteria inhibition (Dabsie et al., 2009; Li et al., 2016). On this matter, Dabsie et al. showed that Sr^2+^ ions have no significant antibacterial effect at the concentrations of 0.19, 0.37, 0.74, and 1.11 mol/L. However, they suggested that Sr^2+^ ions could have a synergistic effect with F^−^ ions in promoting the antibacterial activity.

### *In vitro* Behavior of Sr-Containing Glasses

The biological properties of bivalent cation-doped BGs were reviewed in details elsewhere (Cacciotti, 2017). Regardless of the former oxides used for the design of the glass network, several studies demonstrated that the addition of Sr into the glass composition could promote osteoblast activity and inhibit osteoclasts *in vitro* (Gentleman et al., 2010). It was shown that Sr^2+^ concentrations ranging between 8.7 and 87.6 ppm could induce a positive response of osteoblastic phenotypes (Murphy et al., 2009). Enhanced ALP activity (Hesaraki et al., 2010a), together with higher mineralization (Kargozar et al., 2017) and production of ECM were also reported (Bonnelye et al., 2008; Gentleman et al., 2010; Hesaraki et al., 2010b; Bellucci and Cannillo, 2018). Some other studies pointed out the beneficial effects of Sr release on cellular attachment, proliferation (O'donnell et al., 2010) and differentiation (Isaac et al., 2011; Strobel et al., 2013; Santocildes-Romero et al., 2015; Stefanic et al., 2018).

It was reported that the substitution of Ca with Sr in the 45S5 glass composition led to an altered biological response *in vitro*, as a result of the modifications of the physical and chemical properties (density and solubility). Cellular metabolic activity was critically inhibited by totally replacing Ca with Sr, but a positive effect on mesenchymal stromal cells derived from rat bone marrow was observed in the glass with a molar SrO/CaO substitution of 50% (Santocildes-Romero et al., 2015).

In another study, *in vitro* experiments with osteosarcoma cells revealed that 5 mol% was the optimal Sr concentration in sol-gel BGs for stimulating bone cell production of ALP (Solgi et al., 2017).

The cytocompatibility of CaO–SrO–Na_2_O–ZnO–SiO_2_ glass systems was compared to that of commercial Novabone® (i.e., 45S5 Bioglass®), used as the control in order to evaluate their potential as synthetic bone grafts. Cell viability assays revealed no significantly increased cell viability when compared to commercial control. Sr^2+^ concentration was found to range between 0.8 and 38 ppm, actually in the active stimulatory range for biomedical applications. Interestingly, it was observed that the beneficial effect on cells was the result of a synergistic combination of the ions dissolved in the culture medium (Murphy et al., 2010), as enhanced proliferation was also observed with otherwise-inactive concentrations of Ca^2+^ and silicate ions only in Sr-doped systems, coherently with another previous study (Wu et al., 2007). Comparable results in terms of cellular proliferation were reported by Hesaraki et al., who observed that the inclusion of SrO within the glass network of silicophosphate glasses promoted cell proliferation and ALP activity depending on time (Hesaraki et al., 2010b).

Naruphontjirakul et al. recently investigated the response of MC3T3-E1 cells to BG nanoparticles (diameter 80-100 nm) doped with different SrO amounts (Naruphontjirakul et al., 2018). No negative effects on *in vitro* cell viability were observed with up to 250 μg/mL of glass, and the ionic dissolution products were non-toxic, regardless of the concentrations. However, concentrations of Sr-doped nanoparticles at or above 500 μg/mL led to cellular death after 3 days when the direct contact testing method was used (Naruphontjirakul et al., 2018). This study interestingly demonstrated that the concentration of glass nanoparticles is potentially able to affect both the mineralization mechanism and the synthesis of new extracellular matrix; enhanced protein expression (ALP, OSC, and OSP) and collagen production were also observed as Sr increased both in the nanoparticle composition and the dissolution products (Naruphontjirakul et al., 2018).

Cytotoxicity evaluation performed by Oudadesse et al. on both Sr-doped and Sr-free glasses revealed no differences related to the Sr addition within the network and its subsequent release in contact with the biological environment (Oudadesse et al., 2011). Inhibition of the pro-inflammatory response was also observed in direct contact testing mode by exposing murine macrophages to 1 mg/mL Sr-doped MBG particles, with a significant reduction of IL6 and IL1β expressions in MBG containing 2 mol% of Sr (Fiorilli et al., 2018).

### *In vivo* Evaluations of Sr-Containing Glasses

The *in vivo* effects of Sr-doped biomaterials in bone formation and remodeling was exhaustively reported by Neves et al. (2017). The *in vivo* bone response to Sr-containing SiO_2_-CaO–Na_2_O–P_2_O_5_ glass particles was first reported by Gorustovich et al. in 2009 (Gorustovich et al., 2010). This study revealed no remarkable differences in terms of osteoconductivity with respect to the 45S5 glass control system. In fact, the amount of Sr detected at the interface did not alter the osteoconductive properties, and Sr was not found in the newly-formed bone tissue.

In the same year, Boyd et al. (2009) designed a bone graft with composition 28SrO−32ZnO−40SiO_2_ (mol%) and implanted it in a standard rat femur model using healthy Wistar rats. No inflammatory response was detected, and the same material was then implanted into ovariectomized rats in order to evaluate the therapeutic potential in osteoporotic bone. The performance of the material was found to be almost the same both in healthy and osteoporotic bone, which was encouraging as a bone response to implants is usually diminished in ovariectomized rats and, therefore, suggested the great potential of such material in the treatment of osteoporosis.

Antioxidant properties and regenerative bone capacity were also investigated by Jebahi et al. *in vivo*. Sr-doped glasses were implanted in the femoral condyles of Wistar rats; experimental results showed a remarkable improvement in cell proliferation and antioxidant properties against reactive oxygen species (ROSs) due to three different mechanisms: (i) physiological bone remodeling enhanced by the ions released which supported cells to restore their physiological functionality and enhancement of osteoblastic activity, responsible for the production antioxidant species (GPx); (ii) Sr support in the elimination of oxidative radicals, thus protecting the surrounding tissue against damage; (iii) combined action of Sr and other ions released from the material which contributed to restore a balanced oxidative status (Jebahi et al., 2012).

**Figure 11:**
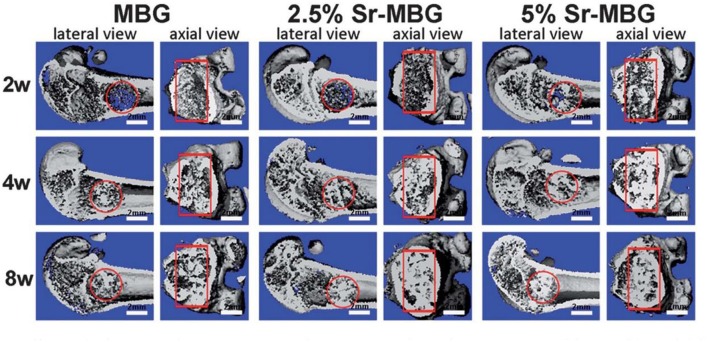
Micro-CT images from implantation of MBGs doped with 2.5 and 5% Sr in the critical femoral defect of ovariectomized rats at two, four, and 8 weeks post-surgery. The red circle and rectangle show the boundary of the defected sites. As shown, a little new bone is present in the defects at 2 weeks, while abundant new bone is observed at the other time points (four and 8 weeks) which depicted visible difference among the groups. Scale bar 2 mm. Adapted from Zhang et al. (2013) with permission by The Royal Society.

Zhang et al. showed that Sr-incorporated MBGs scaffolds could stimulate *in vivo* regeneration of osteoporotic bone defects (Zhang et al., 2013). They implanted the scaffolds in critical-sized femur defects in ovariectomized rats and evaluated the bone regeneration process at time points 2, 4, and 8 weeks post-implantation. The results revealed an enhanced new bone formation in the animals treated with Sr-MBG scaffolds in comparison to those treated with MBG scaffolds alone (See Figure 11). The authors stated that Sr-MBG scaffolds could be a promising candidate for regenerating osteoporosis-related injuries. Moreover, O'Connell et al. (2017) observed a reduction in the inflammatory cell infiltration, but no significant differences in terms of neovascularization and fibrosis. These results indicate that Sr has no effects on the normal healing process, thus confirming the enormous potential of Sr-doped materials in biomedical applications such as bone augmentation. However, it is worth underlying that recent studies concern about cardiac safety of Sr, for example, in the form of SR used currently as a treatment for patients with osteoporosis (Donneau and Reginster, 2014; Reginster, 2014). Although the increased risk for cardiac events with SR was detected in randomized controlled trials (RCTs), the situation does not appear in real life, and Sr remains a beneficial therapeutic alternative in patients suffering severe osteoporosis without cardiovascular contraindications, who cannot receive another osteoporosis treatment.

## Conclusions and Outlook

Using therapeutic elements released from implantable biomaterials to efficiently and locally treat various diseases is arising as one of the major challenges of modern bioengineering and regenerative medicine. Sr is known to be beneficial to patients who suffer from osteoporosis due to its capability of promoting osteoblast function and inhibiting osteoclast activity. Therefore, Sr incorporation in BGs may be a valuable strategy to deliver a steady supply of Sr^2+^ ions to osseous defect sites in the elderly. However, too much osteoclast inhibition may delay bone remodeling and, hence, tissue regeneration in osteoporotic patients. BG composition, amount of Sr incorporated and form of application (e.g., granules, injectable cements, porous scaffolds) are all key factors that dictate the release kinetics of Sr^2+^ ions and should be taken into account while developing potentially suitable biomaterials for the advanced treatment of osteoporosis.

To the best of the authors' knowledge, only one Sr-containing glass (melt-derived Stronbone^TM^, 44.5SiO_2_−4Na_2_O−4K_2_O−7.5MgO−17.8CaO−4.4P_2_O_5_−17.8SrO mol%) achieved the market as well as clinical applications in the form of particles and granules for dental applications (periodontal defect filler and toothpastes) (Hill and Stevens, 2009). More recently, it was shown that Sr could be easily incorporated in sol-gel systems, too. In this regard, MBGs might represent the future of Sr-doped glass-based biomaterials being able to allow the finely-controlled release of various therapeutic metallic elements (Kargozar et al., 2018a), including Sr. Compared to melt-derived glasses, these sol-gel materials potentially exhibit higher versatility in terms of ion release rate, which could ideally be tailored according to the needs of each patient, or clinical case.

Furthermore, there is early evidence suggesting the antibacterial potential of Sr^2+^ ions. The molecular mechanisms behind the antiseptic activity of Sr should still be well-elucidated, but this could open new horizons toward the development of multifunctional Sr-releasing BGs able to promote bone regeneration and prevent infections, thus significantly accelerating the healing of bone injuries.

## Author Contributions

SK, MM, EF, and FB: wrote the first draft. SK, MM, and FB: prepared the revisions.

### Conflict of Interest Statement

The authors declare that the research was conducted in the absence of any commercial or financial relationships that could be construed as a potential conflict of interest.

## References

[B1] Abou Neel E. A. Chrzanowski W. Pickup D. M. O'Dell L. A. Mordan N. J. Newport M. E.. (2009). Structure and properties of strontium-doped phosphate-based glasses. J. Royal Soc. Interface 6, 435–446. 10.1098/rsif.2008.0348 18826914 PMC2659698

[B2] Agarwal S. Wendorff J. H Greiner A. (2008). Use of electrospinning technique for biomedical applications. Polymer 49, 5603–5621. 10.1016/j.polymer.2008.09.014

[B3] Aimaiti A. Maimaitiyiming A. Boyong X. Aji K. Li C. Cui L. (2017). Low-dose strontium stimulates osteogenesis but high-dose doses cause apoptosis in human adipose-derived stem cells via regulation of the ERK1/2 signaling pathway. Stem Cell Res. Ther. 8:282. 10.1186/s13287-017-0726-8 29254499 PMC5735894

[B4] Aina V. Bergandi L. Lusvardi G. Malavasi G. Imrie F. E. Gibson G.. (2013). Sr-containing hydroxyapatite: morphologies of HA crystals and bioactivity on osteoblast cells. Mater. Sci. Eng. 33, 1132–1142. 10.1016/j.msec.2012.12.005 23827552

[B5] Baino F. (2017). Ceramics for bone replacement: commercial products, and clinical use, in Advances in Ceramic Biomaterials, eds Palmero P. Cambier F. De Barra E. (Cambridge: Elsevier), 249–278. 10.1016/B978-0-08-100881-2.00007-5

[B6] Baino F. Fiorilli S. Vitale-Brovarone C. (2016). Bioactive glass-based materials with hierarchical porosity for medical applications: review of recent advances. Acta Biomater. 42, 18–32. 10.1016/j.actbio.2016.06.033 27370907

[B7] Baino F. Fiume E. Barberi J. Kargozar S. Marchi J. Massera J. (2019). Processing methods for making porous bioactive glass-based scaffolds—A state-of-the-art review. Int. J. Appl. Ceramic Technol. 10.1111/ijac.13195. [Epub ahead of print].

[B8] Baino F. Fiume E. Miola M. Verné E. (2018). Bioactive sol-gel glasses: processing, properties, and applications. Int. J. Appl. Ceramic Technol. 15, 841–860. 10.1111/ijac.12873

[B9] Baino F. Hamzehlou S. Kargozar S. (2018a). Bioactive glasses: where are we and where are we going? J. Funct. Biomater. 9:25. 10.3390/jfb9010025 29562680 PMC5872111

[B10] Balasubramanian P. Buettner T. Pacheco V. M. Boccaccini A. R. (2018). Boron-containing bioactive glasses in bone and soft tissue engineering. J. Eur. Ceramic Soc. 38, 855–869. 10.1016/j.jeurceramsoc.2017.11.001

[B11] Baron R. Tsouderos Y. (2002). *In vitro* effects of S12911-2 on osteoclast function and bone marrow macrophage differentiation. Eur. J. Pharmacol. 450, 11–17. 10.1016/S0014-2999(02)02040-X 12176103

[B12] Bauer J. Carvalho E. M. Carvalho C. N. Meier M. M. de Souza J. P. de Carvalho R. M. (2016). Development of a simplified etch-and-rinse adhesive containing niobiophosphate bioactive glass. Int. J. Adh. Adhes. 69, 110–114. 10.1016/j.ijadhadh.2016.03.015

[B13] Bellucci D. Cannillo V. (2018). A novel bioactive glass containing strontium and magnesium with ultra-high crystallization temperature. Mater. Lett. 213, 67–70. 10.1016/j.matlet.2017.11.020

[B14] Bellucci D. Sola A. Cacciotti I. Bartoli C. Gazzarri M. Bianco A.. (2014). Mg-and/or Sr-doped tricalcium phosphate/bioactive glass composites: Synthesis, microstructure and biological responsiveness. Mater. Sci. Eng. C 42, 312–324. 10.1016/j.msec.2014.05.047 25063124

[B15] Bellucci D. Sola A. Salvatori R. Anesi A. Chiarini L. Cannillo V. (2017). Role of magnesium oxide and strontium oxide as modifiers in silicate-based bioactive glasses: effects on thermal behaviour, mechanical properties and *in-vitro* bioactivity. Mater. Sci. Eng. C 72, 566–575. 10.1016/j.msec.2016.11.110 28024623

[B16] Bhambri S. K. Gilbertson L. N. (1995). Micromechanisms of fatigue crack initiation and propagation in bone cements. J. Biomed. Mater. Res. 29, 233–237. 10.1002/jbm.820290214 7738071

[B17] Bohner M. (2000). Calcium orthophosphates in medicine: from ceramics to calcium phosphate cements, Injury 31, D37–D47. 10.1016/S0020-1383(00)80022-4 11270080

[B18] Bonnelye E. Chabadel A. Saltel F. Jurdic P. (2008). Dual effect of strontium ranelate: stimulation of osteoblast differentiation and inhibition of osteoclast formation and resorption *in vitro*. Bone 42, 129–138. 10.1016/j.bone.2007.08.043 17945546

[B19] Boyd D. Carroll G. Towler M. Freeman C. Farthing P. Brook I. (2009). Preliminary investigation of novel bone graft substitutes based on strontium–calcium–zinc–silicate glasses. J. Mater. Sci. 20, 413–420. 10.1007/s10856-008-3569-0 18839286

[B20] Boyd D. Towler M. R. Watts S. Hill R. G. Wren A. W. Clarkin O. M. (2008). The role of Sr 2+ on the structure and reactivity of SrO–CaO–ZnO–SiO 2 ionomer glasses. J. Mater. Sci. 19, 953–957. 10.1007/s10856-006-0060-7 17665132

[B21] Boyle W. J. Simonet W. S. Lacey D. L. (2003). Osteoclast differentiation and activation. Nature 423, 337–342. 10.1038/nature01658 12748652

[B22] Brauer D. S. Karpukhina N. Kedia G. Bhat A. Law R. V. Radecka I.. (2012). Bactericidal strontium-releasing injectable bone cements based on bioactive glasses. J. R. Soc. Interface 10:rsif20120647. 10.1098/rsif.2012.0647 23097502 PMC3565794

[B23] Brow R. K. (2000). The structure of simple phosphate glasses. J. Non-Crystal. Solids 263, 1–28. 10.1016/S0022-3093(99)00620-1

[B24] Brown S. D. Martin M. Deshpande S. Seal S. Huang K. Alm E.. (2006). Cellular response of Shewanella oneidensis to strontium stress. Appl. Environ. Microbiol. 72, 890–900. 10.1128/AEM.72.1.890-900.2006 16391131 PMC1352239

[B25] Busuioc M. Mackiewicz K. Buttaro B. A. Piggot P. J. (2009). Role of Intracellular Polysaccharide in Persistence of *Streptococcus mutans*. J. Bacteriol. 191, 7315–7322. 10.1128/JB.00425-09 19801415 PMC2786568

[B26] Cacciotti I. (2017). Bivalent cationic ions doped bioactive glasses: the influence of magnesium, zinc, strontium and copper on the physical and biological properties. J. Mater. Sci. 52, 8812–8831. 10.1007/s10853-017-1010-0

[B27] Cai S. Li J. Xu G. Li X. Ye X. Jiang W. (2011). *In vitro* solubility and bioactivity of Sr and Mg co-doped calcium phosphate glass-ceramics derived from different heat-treatment temperatures. Mater. Chem. Phys. 131, 462–470. 10.1016/j.matchemphys.2011.10.005

[B28] Cao W. Hench L. L. (1996). Bioactive materials. Ceram. Int. 22, 493–507. 10.1016/0272-8842(95)00126-3

[B29] Chen Y. Whetstone H. C. Lin A. C. Nadesan P. Wei Q. Poon R.. (2007). Beta-catenin signaling plays a disparate role in different phases of fracture repair: implications for therapy to improve bone healing. PLoS Med. 4:e249. 10.1371/journal.pmed.0040249 17676991 PMC1950214

[B30] Cheung K. M. Lu W. W. Luk K. D. Wong C. T. Chan D. Shen X.. (2005). Vertebroplasty by use of a strontium-containing bioactive bone cement. Spine 30, S84–S91. 10.1097/01.brs.0000175183.57733.e5 16138071

[B31] Christie J. K. de Leeuw N. H. (2017). Effect of strontium inclusion on the bioactivity of phosphate-based glasses. J. Mater. Sci. 52, 9014–9022. 10.1007/s10853-017-1155-x PMC6991965 32055076

[B32] Clarkin O. Boyd D. Madigan S. Towler M. (2009). Comparison of an experimental bone cement with a commercial control, Hydroset™. J. Mater. Sci. 20, 1563–1570. 10.1007/s10856-009-3701-9 19214713

[B33] Clarkin O. Boyd D. Towler M. (2010). Strontium-based glass polyalkenoate cements for luting applications in the skeleton. J. Biomater. Appl. 24, 483–502. 10.1177/0885328208099085 19074470

[B34] Cui X. Huang C. Zhang M. Ruan C. Peng S. Li L.. (2017). Enhanced osteointegration of poly (methylmethacrylate) bone cements by incorporating strontium-containing borate bioactive glass. J. R. Soc. Interface 14:20161057. 10.1098/rsif.2016.1057 28615491 PMC5493788

[B35] Dabsie F. Gregoire G. Sixou M. Sharrock P. (2009). Does strontium play a role in the cariostatic activity of glass ionomer? strontium diffusion and antibacterial activity. J. Dentistry 37, 554–559. 10.1016/j.jdent.2009.03.013 19410352

[B36] Demian H. W. McDermott K. (1998). Regulatory perspective on characterization and testing of orthopedic bone cements. Biomaterials 19, 1607–1618. 10.1016/S0142-9612(97)00122-1 9830987

[B37] Dessou N. Theodorou G. Kantiranis N. Papadopoulou L. Zorba T. Patsiaoura D. (2017). Influence of strontium for calcium substitution on the glass–ceramic network and biomimetic behavior in the ternary system SiO 2–CaO–MgO. J. Mater. Sci. 52, 8871–8885. 10.1007/s10853-017-0914-z

[B38] Donneau A.-F. Reginster J.-Y. (2014). Cardiovascular safety of strontium ranelate: real-life assessment in clinical practice. Osteoporos Int. 25, 397–398. 10.1007/s00198-013-2583-3 24322477 PMC3906550

[B39] D'Onofrio A. Kent N. Shahdad S. Hill R. (2016). Development of novel strontium containing bioactive glass based calcium phosphate cement. Dental Mater. 32, 703–712. 10.1016/j.dental.2016.03.006 27033459

[B40] Du J. Xiang Y. (2012). Effect of strontium substitution on the structure, ionic diffusion and dynamic properties of 45S5 bioactive glasses. J. Non-Crystal. Solids 358, 1059–1071. 10.1016/j.jnoncrysol.2011.12.114

[B41] Du J. Xiang Y. (2016). Investigating the structure–diffusion–bioactivity relationship of strontium containing bioactive glasses using molecular dynamics based computer simulations. J. Non Crystalline Solids. 432, 35–40. 10.1016/j.jnoncrysol.2015.03.015

[B42] Erol M. Özyuguran A. Özarpat Ö. Küçükbayrak S. (2012). 3D Composite scaffolds using strontium containing bioactive glasses. J. Eur. Ceram. Soc. 32, 2747–2755. 10.1016/j.jeurceramsoc.2012.01.015

[B43] Fagerlund S. Hupa L. (2016). Melt-derived bioactive silicate glasses, in Bioactive Glasses, eds Boccaccini A. R. Brauer D. S. Hupa L. (Cambridge: Royal Society of Chemistry), 1–26.

[B44] Fernandes J. S. Gentile P. Martins M. Neves N. M. Miller C Crawford A.. (2016). Reinforcement of poly-l-lactic acid electrospun membranes with strontium borosilicate bioactive glasses for bone tissue engineering. Acta Biomater. 44, 168–177. 10.1016/j.actbio.2016.08.042 27554018

[B45] Fiorilli S. Molino G. Pontremoli C. Iviglia G. Torre E. Cassinelli C.. (2018). The incorporation of strontium to improve bone-regeneration ability of mesoporous bioactive glasses. Materials 11:E678. 10.3390/ma11050678 29701683 PMC5978055

[B46] Fiume E. Barberi J. Verné E. Baino F. (2018). Bioactive glasses: from parent 45S5 composition to scaffold-assisted tissue-healing therapies. J. Funct. Biomater. 9:24. 10.3390/jfb9010024 29547544 PMC5872110

[B47] Fredholm Y. C. Karpukhina N. Brauer D. S. Jones J. R. Law R. V. Hill R. G. (2011). Influence of strontium for calcium substitution in bioactive glasses on degradation, ion release and apatite formation. J. R. Soc. Interface 9, 880–889. 10.1098/rsif.2011.0387 21993007 PMC3306632

[B48] Fredholm Y. C. Karpukhina N. Law R. V. Hill R. G. (2010). Strontium containing bioactive glasses: glass structure and physical properties. J. Non-Cryst. Solids 356, 2546–2551. 10.1016/j.jnoncrysol.2010.06.078

[B49] Gentleman E. Fredholm Y. C. Jell G. Lotfibakhshaiesh N. O'Donnell M. D. Hill R. G.. (2010). The effects of strontium-substituted bioactive glasses on osteoblasts and osteoclasts in vitro. Biomaterials 31, 3949–3956. 10.1016/j.biomaterials.2010.01.121 20170952

[B50] Goel A. Rajagopal R. R. Ferreira J. M. (2011). Influence of strontium on structure, sintering and biodegradation behaviour of CaO–MgO–SrO–SiO2–P2O5–CaF2 glasses. Acta Biomater. 7, 4071–4080. 10.1016/j.actbio.2011.06.047 21763793

[B51] Goller G. Demirkiran H. Oktar F. N Demirkesen E. (2003). Processing and characterization of bioglass reinforced hydroxyapatite composites. Ceram. Int. 29, 721–724. 10.1016/S0272-8842(02)00223-7

[B52] Goñi I. Rodríguez R. García-Arnáez I. Parra J. Gurruchaga M. (2018). Preparation and characterization of injectable PMMA-strontium-substituted bioactive glass bone cement composites. J. Biomed. Mater. Res. B Appl. Biomater. 106, 1245–1257. 10.1002/jbm.b.33935 28580716

[B53] Gorustovich A. A. Steimetz T. Cabrini R. L. Porto López J. M. (2010). Osteoconductivity of strontium-doped bioactive glass particles: A histomorphometric study in rats. J. Biomed. Mat. Res. A 92, 232–237. 10.1002/jbm.a.32355 19172615

[B54] Hasan M. S. Werner-Zwanziger U. Boyd D. (2015). Composition-structure-properties relationship of strontium borate glasses for medical applications. J. Biomed. Mater. Res. Part A, 103, 2344–2354. 10.1002/jbm.a.35361 25366812

[B55] He F. Tian Y. (2018). Improvements in phase stability and densification of β-tricalcium phosphate bioceramics by strontium-containing phosphate-based glass additive. Ceram. Int. 44, 11622–11627. 10.1016/j.ceramint.2018.03.236

[B56] Hench L. L. (1991). Bioceramics: from concept to clinic. J. Am. Ceram. Soc. 74, 1487–510. 10.1111/j.1151-2916.1991.tb07132.x

[B57] Hench L. L. (2006). The story of bioglass^®^. J. Mater. Sci. 17, 967–978. 10.1007/s10856-006-0432-z 17122907

[B58] Hench L. L. (2009). Genetic design of bioactive glass. J. Eur. Ceram. Soc. 29, 1257–1265. 10.1016/j.jeurceramsoc.2008.08.002

[B59] Hench L. L. (2013). An Introduction to Bioceramics, 2nd Edn. (London: Imperial College Press).

[B60] Hench L. L. West J. K. (1990). The sol-gel process. Chem. Rev. 90, 33–72. 10.1021/cr00099a003

[B61] Hesaraki S. Alizadeh M. Nazarian H. Sharifi D. (2010b). Physico-chemical and *in vitro* biological evaluation of strontium/calcium silicophosphate glass. J. Mater. Sci. 21, 695–705. 10.1007/s10856-009-3920-0 19866346

[B62] Hesaraki S. Gholami M. Vazehrad S. Shahrabi S. (2010a). The effect of Sr concentration on bioactivity and biocompatibility of sol–gel derived glasses based on CaO–SrO–SiO2–P2O5 quaternary system. Mat. Sci. Eng. 30, 383–390. 10.1016/j.msec.2009.12.001

[B63] Hesaraki S. Hasan Barounian M. Farhangdoust S. Khorami M. Zamanian A. Borhan S. (2012). Mechanical and *in vitro* biological properties of hydroxyapatite bioceramics reinforced with strontium-containing nano-bioactive glass. Curr. Nanosci. 8, 612–622. 10.2174/157341312801784285

[B64] Hill R. Stamboulis A. Law R. Clifford A. Towler M. Crowley C. (2004). The influence of strontium substitution in fluorapatite glasses and glass-ceramics. J. Non Crystal. Solids 336, 223–229. 10.1016/j.jnoncrysol.2004.02.005

[B65] Hill R. G. Stevens M. M. (2009). Bioactive Glass.” in Google Patents.

[B66] Hodges R. M. MacDonald N. S. Nusbaum R. Stearns R. Ezmirlian F. Spain P.. (1950). The strontium content of human bones. J. Biol. Chem. 185, 519–524. 14774392

[B67] Holzwarth J. M. Ma P. X. (2011). Biomimetic nanofibrous scaffolds for bone tissue engineering. Biomaterials 32, 9622–9629. 10.1016/j.biomaterials.2011.09.009 21944829 PMC3195926

[B68] Hoppe A. Güldal N. S. Boccaccini A. R. (2011). A review of the biological response to ionic dissolution products from bioactive glasses and glass-ceramics. Biomaterials 32, 2757–2774. 10.1016/j.biomaterials.2011.01.004 21292319

[B69] Hoppe A. Sarker B. Detsch R. Hild N. Mohn D. Stark W. (2014). *In vitro* reactivity of Sr-containing bioactive glass (type 1393) nanoparticles. J. Non Crystal. Solids 387, 41–46. 10.1016/j.jnoncrysol.2013.12.010

[B70] Huang M. Hill R. G Rawlinson S. C. (2016). Strontium (Sr) elicits odontogenic differentiation of human dental pulp stem cells (hDPSCs): a therapeutic role for Sr in dentine repair? Acta Biomater. 38, 201–211. 10.1016/j.actbio.2016.04.037 27131573

[B71] Hurtel-Lemaire A. S. Mentaverri R. Caudrillier A. Cournarie F. Wattel A. Kamel S.. (2009). The calcium-sensing receptor is involved in strontium ranelate-induced osteoclast apoptosis new insights into the associated signaling pathways. J. Biol. Chem. 284, 575–584. 10.1074/jbc.M801668200 18927086

[B72] Isaac J. Nohra J. Lao J. Jallot E. Nedelec J.-M. Berdal A.. (2011). Effects of strontium-doped bioactive glass on the differentiation of cultured osteogenic cells. Eur. Cell Mater. 21, 130–43. 10.22203/eCM.v021a11 21305476

[B73] Izquierdo-Barba I. Vallet-Regí M. (2015). Mesoporous bioactive glasses: relevance of their porous structure compared to that of classical bioglasses. Biomed. Glasses 1, 140–150. 10.1515/bglass-2015-0014

[B74] Jalise S. Z. Baheiraei N. Bagheri F. (2018). The effects of strontium incorporation on a novel gelatin/bioactive glass bone graft: *in vitro* and *in vivo* characterization. Ceram. Int. 44, 14217–14227. 10.1016/j.ceramint.2018.05.025

[B75] Jebahi S. Oudadesse H. El Feki H. Rebai T. Keskes H. Pellen P. (2012). Antioxidative/oxidative effects of strontium-doped bioactive glass as bone graft. *In vivo* assays in ovariectomised rats. J. Appl. Biomed. 10, 195–209. 10.2478/v10136-012-0009-8

[B76] Jell G. Stevens M. M. (2006). Gene activation by bioactive glasses. J. Mater. Sci. 17, 997–1002. 10.1007/s10856-006-0435-9 17122910

[B77] Johari B. Kadivar M. Lak S. Gholipourmalekabadi M. Urbanska A. M. Mozafari M.. (2016). Osteoblast–seeded bioglass/gelatin nanocomposite: a promising bone substitute in critical-size calvarial defect repair in rat. Int. J. Artif. Organs 39, 524–533. 10.5301/ijao.5000533 27901555

[B78] Jones J. R. (2015). Reprint of: review of bioactive glass: from Hench to hybrids. Acta Biomater. 23, S53–82. 10.1016/j.actbio.2015.07.019 26235346

[B79] Jones J. R. Brauer D. S. Hupa L. Greenspan D. C. (2016). Bioglass and bioactive glasses and their impact on healthcare. Int. J. Appl. Glass Sci. 7, 423–434. 10.1111/ijag.12252

[B80] Kamitakahara M. Ohtsuki C. Miyazaki T. (2008). Behavior of ceramic biomaterials derived from tricalcium phosphate in physiological condition. J. Biomater. Applic. 23, 197–212. 10.1177/0885328208096798 18996965

[B81] Kapoor S. Goel A. Pascual M. J. Ferreira J. M. (2013). Thermo-mechanical behaviour of alkali free bioactive glass-ceramics co-doped with strontium and zinc. J. Non Crystall. Solids 375, 74–82. 10.1016/j.jnoncrysol.2013.05.007

[B82] Kapoor S. Goel A. Tilocca A. Dhuna V. Bhatia G. Dhuna K. M.. (2014). Role of glass structure in defining the chemical dissolution behavior, bioactivity and antioxidant properties of zinc and strontium co-doped alkali-free phosphosilicate glasses. Acta Biomater. 10, 3264–3278. 10.1016/j.actbio.2014.03.033 24709542

[B83] Kargozar S. Baino F. Hamzehlou S. Hill R. G. Mozafari M. (2018b). Bioactive glasses entering the mainstream. Drug Discov. Today, 23, 1700–1704. 10.1016/j.drudis.2018.05.027 29803626

[B84] Kargozar S. Baino F. Hamzehlou S. Hill R. G. Mozafari M. (2018d). Bioactive glasses: Sprouting angiogenesis in tissue engineering. Trends Biotechnol. 36, 430–444. 10.1016/j.tibtech.2017.12.003 29397989

[B85] Kargozar S. Hamzehlou S. Baino F. (2019b). Can bioactive glasses be useful to accelerate the healing of epithelial tissues? Mater. Sci. Eng. C 97, 1009–1020. 10.1016/j.msec.2019.01.028 30678892

[B86] Kargozar S. Lotfibakhshaiesh N. Ai J. Mozafari M. Milan P. B Hamzehlou S.. (2017). Strontium-and cobalt-substituted bioactive glasses seeded with human umbilical cord perivascular cells to promote bone regeneration via enhanced osteogenic, and angiogenic activities. Acta Biomater. 58, 502–514. 10.1016/j.actbio.2017.06.021 28624656

[B87] Kargozar S. Lotfibakhshaiesh N. Ai J. Samadikuchaksaraie A. Hill R. G. Shah P. A. (2016). Synthesis, physico-chemical and biological characterization of strontium and cobalt substituted bioactive glasses for bone tissue engineering. J. Non-Cryst. Solids 449, 133–140. 10.1016/j.jnoncrysol.2016.07.025

[B88] Kargozar S. Montazerian M. Hamzehlou S. Kim H.-W. Baino F. (2018a). Mesoporous bioactive glasses: Promising platforms for antibacterial strategies. Acta Biomater. 81, 1–19. 10.1016/j.actbio.2018.09.052 30273742

[B89] Kargozar S. Mozafari M. (2018). Nanotechnology and Nanomedicine: start small, think big. Mater. Today 5 (7 Part 3), 15492–15500. 10.1016/j.matpr.2018.04.155

[B90] Kargozar S. Mozafari M. Hamzehlou S. Baino F. (2019c). Using bioactive glasses in the management of burns. Front. Bioeng. Biotechnol. 7:62. 10.3389/fbioe.2019.00062 30984751 PMC6447657

[B91] Kargozar S. Mozafari M. Hamzehlou S. Brouki Milan P.-H. Kim W. Baino F. (2019d). Bone tissue engineering using human cells: a comprehensive review on recent trends, current prospects, and recommendations. Appl. Sci. 9:174 10.3390/app9010174

[B92] Kargozar S. Mozafari M. Hamzehlou S.-H. Kim W. Baino F. (2019a). Mesoporous bioactive glasses (MBGs) in cancer therapy: full of hope and promise. Mater. Lett. 251, 241–246. 10.1016/j.matlet.2019.05.019

[B93] Kargozar S. Mozafari M. Hill R. G. Brouki Milan P. Taghi Joghataei M. Hamzehlou S. (2018c). Synergistic combination of bioactive glasses and polymers for enhanced bone tissue regeneration. Mater. Today 5 (7, Part 3), 15532–15539. 10.1016/j.matpr.2018.04.160

[B94] Kenny S. Buggy M. (2003). Bone cements and fillers: a review. J. Mater. Sci. 14, 923–938. 10.1023/A:1026394530192 15348504

[B95] Kent N. W. Hill R. G. Karpukhina N. (2016). A new way of forming a calcium phosphate cement using bioactive glasses as a reactive precursor. Mater. Lett. 162, 32–36. 10.1016/j.matlet.2015.09.099

[B96] Kim H.-W. Koh Y.-H. Kong Y.-M. Kang J.-G. Kim H.-E. (2004). Strontium substituted calcium phosphate biphasic ceramics obtained by a powder precipitation method. J. Mater. Sci. 15, 1129–1134. 10.1023/B:JMSM.0000046395.76435.60 15516874

[B97] Kuang G. M. Yau W. Lam W. Wu J. Chiu K. Lu W. W.. (2012). An effective approach by a chelate reaction in optimizing the setting process of strontium-incorporated calcium phosphate bone cement. J. Biomed. Mater. Res. Part B. 100, 778–787. 10.1002/jbm.b.32511 22331835

[B98] Kuda O. Pinchuk N. Bykov O. Tomila T. Olifan O. Golovkova M. (2018). Development and characterization of Sr-containing glass-ceramic composites based on biogenic hydroxyapatite. Nanoscale Res. Lett. 13l:155 10.1186/s11671-018-2550-1 PMC5955871 29767304

[B99] Kumari C. V. Sobhanachalam P. Jayasankar C. K. Veeraiah N. Kumar V. R. (2017). Bioactive properties of CuO doped CaF2–CaO–B2O3–P2O5–MO(M = Ba, Sr, Zn, Mg) glasses. Ceram. Int. 43, 4335–4343. 10.1016/j.ceramint.2016.12.078

[B100] Lao J. Jallot E. Nedelec J.-M. (2008). Strontium-delivering glasses with enhanced bioactivity: a new biomaterial for antiosteoporotic applications? Chem. Mater. 20, 4969–4973. 10.1021/cm800993s

[B101] Lao J. Lacroix J. Nohra J. Naaman N. Sautier J. (2013). Bioavailability of strontium ions from bioactive glasses *in vivo*: a micro-PIXE study of trace elements at the bone interface. Bioceram. Dev. Appl. 1:1–3. 10.4172/2090-5025.S1-004

[B102] Leite A. J. Gonçalves A. I. Rodrigues M. T. Gomes M. E. Mano J. F. (2018). Strontium-doped bioactive glass nanoparticles in osteogenic commitment. ACS Appl. Mater. Interfaces 10, 23311–23320. 10.1021/acsami.8b06154 29906095

[B103] Li X. Wang X. Chen H. Jiang P. Dong X. Shi J. (2007). Hierarchically porous bioactive glass scaffolds synthesized with a PUF and P123 cotemplated approach. Chem. Mater. 19, 4322–4326. 10.1021/cm0708564

[B104] Li Y. Coughlan A. Wren A. W. (2014). Investigating the surface reactivity of SiO 2–TiO 2–CaO–Na 2 O/SrO bioceramics as a function of structure and incubation time in simulated body fluid. J. Mater. Sci. 25, 1853–1864. 10.1007/s10856-014-5229-x 24796627

[B105] Li Y. Placek L. Coughlan A. Laffir F. Pradhan D. Mellott N. (2015). Investigating the influence of Na+ and Sr 2+ on the structure and solubility of SiO 2–TiO 2–CaO–Na 2 O/SrO bioactive glass. J. Mater. Sci. 26:85 10.1007/s10856-015-5415-5 25644099

[B106] Li Y. Stone W. Schemitsch E. H Zalzal P. Papini M. Waldman S. D.. (2016). Antibacterial and osteo-stimulatory effects of a borate-based glass series doped with strontium ions. J. Biomater. Applicat. 31, 674–683. 10.1177/0885328216672088 27671104

[B107] Liu J. Rawlinson S. C. Hill R. G. Fortune F. (2016). Strontium-substituted bioactive glasses *in vitro* osteogenic and antibacterial effects. Dental Mater. 32, 412–422. 10.1016/j.dental.2015.12.013 26777094

[B108] Lotfibakhshaiesh N. Brauer D. S. Hill R. G. (2010). Bioactive glass engineered coatings for Ti6Al4V alloys: influence of strontium substitution for calcium on sintering behaviour. J. Non Crystal. Solids 356, 2583–2590. 10.1016/j.jnoncrysol.2010.05.017

[B109] Mao L. Xia L. Chang J. Liu J. Jiang L. Wu C.. (2017). The synergistic effects of Sr and Si bioactive ions on osteogenesis, osteoclastogenesis and angiogenesis for osteoporotic bone regeneration. Acta Biomater. 61, 217–232. 10.1016/j.actbio.2017.08.015 28807800

[B110] Marghussian V. (2015). Nano-Glass Ceramics: Processing, Properties and Applications (Oxford: Elsevier).

[B111] Marie P. J. (2007). Strontium ranelate: new insights into its dual mode of action. Bone 40, S5–S8. 10.1016/j.bone.2007.02.003

[B112] Massera J. Petit L. Cardinal T. J.-,Videau J. Hupa M. Hupa L. (2013). Thermal properties and surface reactivity in simulated body fluid of new strontium ion-containing phosphate glasses. J. Mater. Sci. 24, 1407–1416. 10.1007/s10856-013-4910-9 23512152

[B113] Mendez J. Fernández M. Gonzalez-Corchon A. Salvado M. Collia F. De Pedro J.. (2004). Injectable self-curing bioactive acrylic-glass composites charged with specific anti-inflammatory/analgesic agent, Biomaterials 25, 2381–2392. 10.1016/j.biomaterials.2003.09.004 14741603

[B114] Miola M. Pakzad Y. Banijamali S. Kargozar S. Vitale-Brovarone C. Yazdanpanah A. (2019). Glass-ceramics for cancer treatment: So close, or yet so far? Acta Biomater. 83, 55–70. 10.1016/j.actbio.2018.11.013 30415065

[B115] Miola M. Verné E. Ciraldo F. E. Cordero-Arias L. Boccaccini A. R. (2015). Electrophoretic deposition of chitosan/45S5 bioactive glass composite coatings doped with Zn and Sr. Front. Bioeng. Biotechnol. 3:159. 10.3389/fbioe.2015.00159 26539431 PMC4609893

[B116] Misra S. K. Valappil S. P. Roy I. Boccaccini A. R. (2006). Polyhydroxyalkanoate (PHA)/inorganic phase composites for tissue engineering applications. Biomacromolecules 7, 2249–2258. 10.1021/bm060317c 16903667

[B117] Molino G. Bari A. Baino F. Fiorilli S. Vitale-Brovarone C. (2017). Electrophoretic deposition of spray-dried Sr-containing mesoporous bioactive glass spheres on glass–ceramic scaffolds for bone tissue regeneration. J. Mater. Sci. 52, 9103–9114. 10.1007/s10853-017-1026-5

[B118] Montalbano G. Fiorilli S. Caneschi A. Vitale-Brovarone C. (2018). Type I collagen and strontium-containing mesoporous glass particles as hybrid material for 3D printing of bone-like materials. Materials 11:E700. 10.3390/ma11050700 29710811 PMC5978077

[B119] Montazerian M. Yekta B. E. Marghussian V. K. Bellani C. F. Siqueira R, L. Zanotto E. D. (2015). Bioactivity and cell proliferation in radiopaque gel-derived CaO–P 2 O 5–SiO 2–ZrO 2 glass and glass–ceramic powders. Mater. Sci. Eng. 55, 436–447. 10.1016/j.msec.2015.05.065 26117775

[B120] Montazerian M. Zanotto D. (2016). History, and trends of bioactive glass-ceramics. J. Biomed. Mater. Res. A 104, 1231–1249. 10.1002/jbm.a.35639 26707951

[B121] Montazerian M. Zanotto E. D. (2016). Bioactive glass-ceramics: processing, properties and applications. Bioactive Glasses 27–33. 10.1039/9781782622017-00027

[B122] Montazerian M. Zanotto E. D. (2017a). A guided walk through Larry Hench's monumental discoveries. J. Mater. Sci. 52, 8695–8732. 10.1007/s10853-017-0804-4

[B123] Montazerian M. Zanotto E. D. (2017b). Bioactive and inert dental glass-ceramics. J. Biomed. Mater. Res. A 105, 619–639. 10.1002/jbm.a.35923 27701809

[B124] Mozafari M. Banijamali S. Baino F. Kargozar S. Hill R. G. (2019). Calcium carbonate: adored and ignored in bioactivity assessment. Acta Biomater. 91, 35–47. 10.1016/j.actbio.2019.04.039 31004843

[B125] Murphy S. Boyd D. Moane S. Bennett M. (2009). The effect of composition on ion release from Ca–Sr–Na–Zn–Si glass bone grafts. J. Mater. Sci. 20, 2207. 10.1007/s10856-009-3789-y 19475338

[B126] Murphy S. Wren A. Towler M. Boyd D. (2010). The effect of ionic dissolution products of Ca–Sr–Na–Zn–Si bioactive glass on *in vitro* cytocompatibility. J. Mater. Sci. 21, 2827–2834. 10.1007/s10856-010-4139-9 20711638

[B127] Naruphontjirakul P. Porter A. E. Jones J. R. (2018). *In vitro* osteogenesis by intracellular uptake of strontium containing bioactive glass nanoparticles. Acta Biomater. 66, 67–80. 10.1016/j.actbio.2017.11.008 29129790

[B128] Neves N. Linhares D. Costa G. Ribeiro C. Barbosa M. (2017). *In vivo* and clinical application of strontium-enriched biomaterials for bone regeneration: a systematic review. Bone Joint Res. 6, 366–375. 10.1302/2046-3758.66.BJR-2016-0311.R1 28600382 PMC5492369

[B129] Newman S. D. Lotfibakhshaiesh N. O'Donnell M. Walboomers X. F Horwood N. Jansen J. A.. (2014). Enhanced osseous implant fixation with strontium-substituted bioactive glass coating. Tissue Eng. A 20, 1850–1857. 10.1089/ten.tea.2013.0304 24471799

[B130] Nezafati N. Hesaraki S. Badr-Mohammadi M.-R. (2014). Synthesis, characterization and *in vitro* evaluation of strontium-containing sol-gel derived bioactive glass/biphasic calcium phosphate nanocomposite. Appl. Mech. Mater. 467, 64–69. 10.4028/www.scientific.net/AMM.467.64

[B131] Ni G. Lu W. Chiu K. Li Z. Fong D. Luk K. (2006). Strontium-containing hydroxyapatite (Sr-HA) bioactive cement for primary hip replacement: an *in vivo* study. J. Biomed. Mater. Res. Part B. 77, 409–415. 10.1002/jbm.b.30417 16278857

[B132] Niinomi M. (2010). Metals for Biomedical Devices (Cambridge: Elsevier). 10.1533/9781845699246

[B133] O'Brien D. Boyd D. Madigan S. Murphy S. (2010). Evaluation of a novel radiopacifiying agent on the physical properties of surgical spineplex®. J Mat Sci. 21, 53–58. 10.1007/s10856-009-3844-8 19688251

[B134] O'Connell K. Pierlot C. O'Shea H. Beaudry D. Chagnon M. Assad M.. (2017). Host responses to a strontium releasing high boron glass using a rabbit bilateral femoral defect model. J. Biomed. Mater. Res. B 105, 1818–1827. 10.1002/jbm.b.33694 27219680

[B135] O'donnell M. Candarlioglu P. Miller C. Gentleman E. Stevens M. (2010). Materials characterisation and cytotoxic assessment of strontium-substituted bioactive glasses for bone regeneration. J. Mater. Chem. 20, 8934–8941. 10.1039/c0jm01139h

[B136] O'donnell M. Hill R. (2010). Influence of strontium and the importance of glass chemistry and structure when designing bioactive glasses for bone regeneration. Acta Biomater. 6, 2382–2385. 10.1016/j.actbio.2010.01.006 20079468

[B137] O'Donnell S. Cranney A. Wells G. A. Adachi J. D Reginster J. Y (2006). Strontium ranelate for preventing and treating postmenopausal osteoporosis. Cochr. Database Syst. Rev. 10.1002/14651858.CD005326.pub2 16856092

[B138] Omar S. Repp F. Desimone P. M. Weinkamer R. Wagermaier W. Ceré S. (2015). Sol–gel hybrid coatings with strontium-doped 45S5 glass particles for enhancing the performance of stainless steel implants: electrochemical, bioactive and *in vivo* response. J. Non Crystall. Solids 425, 1–10. 10.1016/j.jnoncrysol.2015.05.024

[B139] Oudadesse H. Dietrich E. Bui X. V. Le Gal Y. Pellen P. Cathelineau G. (2011). Enhancement of cells proliferation and control of bioactivity of strontium doped glass. Appl. Surf. Sci. 257, 8587–8593. 10.1016/j.apsusc.2011.05.022

[B140] Owens G. J. Singh R. K. Foroutan F. Alqaysi M. Han C.-M. Mahapatra C. (2016). Sol–gel based materials for biomedical applications. Progr. Mater. Sci. 77, 1–79. 10.1016/j.pmatsci.2015.12.001

[B141] Özarslan A. C. Yücel S. (2016). Fabrication, and characterization of strontium incorporated 3-D bioactive glass scaffolds for bone tissue from biosilica. Mater. Sci. Eng. C 68, 350–357. 10.1016/j.msec.2016.06.004 27524030

[B142] Pan H. Zhao X. Zhang X. Zhang K. Li L. Li Z.. (2009). Strontium borate glass: potential biomaterial for bone regeneration. J. R. Soc. Interface 7:rsif 20090504. 10.1098/rsif.2009.0504 20031984 PMC2880081

[B143] Patel U. Moss R. Hossain K. M. Z. Kennedy A. R. Barney E. R. Hannon A. C.. (2017). Structural and physico-chemical analysis of calcium/strontium substituted, near-invert phosphate based glasses for biomedical applications. Acta Biomater. 60, 109–127. 10.1016/j.actbio.2017.07.002 28684335

[B144] Peng S. Zhou G. Luk K. D. Cheung K. M. Li Z. Lam Z.. (2009). Strontium promotes osteogenic differentiation of mesenchymal stem cells through the Ras/MAPK signaling pathway. Cell. Physiol. Biochem. 23, 165–174. 10.1159/000204105 19255511

[B145] Pina S. Torres P. Goetz-Neunhoeffer F. Neubauer J. Ferreira J. (2010). Newly developed Sr-substituted α-TCP bone cements. Acta Biomater. 6, 928–935. 10.1016/j.actbio.2009.09.001 19733700

[B146] Piotrowski G. Hench L. Allen W. Miller G. (1975). Mechanical studies of the bone bioglass interfacial bond. J. Biomed. Mater. Res. 9, 47–61. 10.1002/jbm.820090408 809448

[B147] Rahaman M. N. Day D. E. Bal B. S. Fu Q. Jung S. B. Bonewald L. F.. (2011). Bioactive glass in tissue engineering. Acta Biomater. 7, 2355–2373. 10.1016/j.actbio.2011.03.016 21421084 PMC3085647

[B148] Rawlings R. D. (1993). Bioactive glasses, and glass-ceramics. Clin. Mater. 14, 155–179. 10.1016/0267-6605(93)90038-9 10146444

[B149] Reginster J.-Y. (2014). Cardiac concerns associated with strontium ranelate. Expert Opin. Drug Safety 13, 1209–1213. 10.1517/14740338.2014.939169 25020233 PMC4196504

[B150] Ren J. Blackwood K. A. Doustgani A. Poh P. P. Steck R. Stevens M. M.. (2014). Melt-electrospun polycaprolactone strontium-substituted bioactive glass scaffolds for bone regeneration. J. Biomed. Mater. Res. A 102, 3140–3153. 10.1002/jbm.a.34985 24133006

[B151] Rokidi S. Koutsoukos P. G. (2012). Crystal growth of calcium phosphates from aqueous solutions in the presence of strontium. Chem. Eng. Sci. 77, 157–164. 10.1016/j.ces.2012.02.049

[B152] Sabokbar A. Fujikawa Y. Murray D. W. Athanasou N. A. (1997). Radio-opaque agents in bone cement increase bone resorption. J. Bone Joint Surg. 79, 129–134. 10.1302/0301-620X.79B1.6966 9020461

[B153] Saidak Z. Marie P. J. (2012). Strontium signaling: molecular mechanisms and therapeutic implications in osteoporosis. Pharmacol. Ther. 136, 216–226. 10.1016/j.pharmthera.2012.07.009 22820094

[B154] Saint-Jean S. J. Camire C. Nevsten P. Hansen S. Ginebra M. (2005). Study of the reactivity and *in vitro* bioactivity of Sr-substituted α-TCP cements. J. Mater. Sci. 16, 993–1001. 10.1007/s10856-005-4754-z 16388381

[B155] Santocildes-Romero M. E. Crawford A. Hatton P. V Goodchild R. L. Reaney I. M. Miller C. A. (2015). The osteogenic response of mesenchymal stromal cells to strontium-substituted bioactive glasses. J. Tissue Eng. Regenerat. Med. 9, 619–631. 10.1002/term.2003 25757935 PMC5053305

[B156] Santocildes-Romero M. E. Goodchild R. L. Hatton P. V. Crawford A. Reaney I. M. Miller C. A. (2016). Preparation of composite electrospun membranes containing strontium-substituted bioactive glasses for bone tissue regeneration. Macromol. Mater. Eng. 301, 972–981. 10.1002/mame.201600018

[B157] Schindeler A. Little D. G. (2006). Ras-MAPK signaling in osteogenic differentiation: friend or foe? J. Bone Min. Res. 21, 1331–1338. 10.1359/jbmr.060603 16939391

[B158] Schumacher M. Lode A. Helth A. Gelinsky M. (2013). A novel strontium(II)-modified calcium phosphate bone cement stimulates human-bone-marrow-derived mesenchymal stem cell proliferation and osteogenic differentiation *in vitro*. Acta Biomater. 9, 9547–9557. 10.1016/j.actbio.2013.07.027 23917042

[B159] Solgi S. Khakbiz M. Shahrezaee M. Zamanian A. Tahriri M. Keshtkari S. (2017). Synthesis, characterization and *in vitro* biological evaluation of Sol-gel derived Sr-containing nano bioactive glass. Silicon 9, 535–542. 10.1007/s12633-015-9291-x

[B160] Sriranganathan D. Kanwal N. Hing K. A. Hill R. G. (2016). Strontium substituted bioactive glasses for tissue engineered scaffolds: the importance of octacalcium phosphate. J. Mater. Sci. 27:39. 10.1007/s10856-015-5653-6 26704556 PMC4690837

[B161] Stefanic M. Peroglio M. Stanciuc A.-M. Machado G. Campbell I. KrŽmanc M. M. (2018). The influence of strontium release rate from bioactive phosphate glasses on osteogenic differentiation of human mesenchymal stem cells. J. Eur. Ceram. Soc. 38, 887–897. 10.1016/j.jeurceramsoc.2017.08.005

[B162] Strobel L. Hild N. Mohn D. Stark W. J. Hoppe A. Gbureck U. (2013). Novel strontium-doped bioactive glass nanoparticles enhance proliferation and osteogenic differentiation of human bone marrow stromal cells. J. Nanoparticle Res. 15:1780 10.1007/s11051-013-1780-5

[B163] Taherkhani S. Moztarzadeh F. (2016). Influence of strontium on the structure and biological properties of sol–gel-derived mesoporous bioactive glass (MBG) powder. J. Sol Gel Sci. Technol. 78, 539–549. 10.1007/s10971-016-3995-2

[B164] Tallia F. Gallo M. Pontiroli L. Baino F. Fiorilli S. Onida B. (2014). Vitale-Brovarone: zirconia-containing radiopaque mesoporous bioactive glasses. Mater. Lett. 130, 281–284. 10.1016/j.matlet.2014.05.062

[B165] Thormann U. Ray S. Sommer U. ElKhassawna T. Rehling T. Hundgeburth M. Henß A.. (2013). Bone formation induced by strontium modified calcium phosphate cement in critical-size metaphyseal fracture defects in ovariectomized rats. Biomaterials 34, 8589–8598. 10.1016/j.biomaterials.2013.07.036 23906515

[B166] Tilocca A. (2010). Models of structure, dynamics and reactivity of bioglasses: a review. J. Mater. Chem. 20, 6848–6858. 10.1039/c0jm01081b

[B167] Topalović V. S. Grujić S. R. Živanović V. D. Matijašević S. D. Nikolić J. D. Stojanović J. N (2017) Bioactive glass-ceramics prepared by powder sintering crystallization of polyphosphate glass containing strontium. Ceram. Int, 43, 12061–12069. 10.1016/j.ceramint.2017.06.061.

[B168] Vaughan J. M. (1975). The Physiology of Bone. Oxford University Press.

[B169] Verberckmoes S. C. De Broe M. E. D'Haese P. C. (2003). Dose-dependent effects of strontium on osteoblast function, and mineralization. Kidney Int. 64, 534–543. 10.1046/j.1523-1755.2003.00123.x 12846748

[B170] Verné E. (2012). Bioactive glass, and glass-ceramic, coatings, in Bio-Glasses: An Introduction, eds Jones J. R. Clare A. G. (West Sussex: John Wiley & Sons), 107–119. 10.1002/9781118346457.ch8

[B171] Wang J.-S. Diaz J. Sabokbar A. Athanasou N. Kjellson F. Tanner K.. (2005). *In vitro* and *in vivo* biological responses to a novel radiopacifying agent for bone cement. J. R. Soc. Interface 2, 71–78. 10.1098/rsif.2004.0009 16849166 PMC1578263

[B172] Wang X. Li X. Ito A. Sogo Y. (2011). Synthesis and characterization of hierarchically macroporous and mesoporous CaO–MO–SiO2–P2O5 (M = Mg, Zn, Sr) bioactive glass scaffolds. Acta Biomater. 7, 3638–3644. 10.1016/j.actbio.2011.06.029 21742065

[B173] Wegman M. Eisenberg A. Curzon M. Handelman S. (1984). Effects of fluoride, lithium, and strontium on intracellular polysaccharide accumulation in *S. mutans* and *A. viscosu*s. J. Dent. Res. 63, 1126–1129. 10.1177/00220345840630090601 6589274

[B174] Wei L. Ke J. Prasadam I. Miron R. J. Lin S. Xiao J.. (2014). A comparative study of Sr-incorporated mesoporous bioactive glass scaffolds for regeneration of osteopenic bone defects. Osteop. Int. 25, 2089–2096. 10.1007/s00198-014-2735-0 24807629

[B175] Weiss D. Torres R. Buchner S. Blunk S. Soares P. (2014). Effect of Ti and Mg dopants on the mechanical properties, solubility, and bioactivity *in vitro* of a Sr-containing phosphate based glass. J. Non-Crystal. Solids 386, 34–38. 10.1016/j.jnoncrysol.2013.11.036

[B176] Wren A. Boyd D. Towler M. (2008). The processing, mechanical properties and bioactivity of strontium based glass polyalkenoate cements. J. Mater. Sci. 19, 1737–1743. 10.1007/s10856-007-3287-z 17943414

[B177] Wren A. Coughlan A. Hall M. German M. Towler M. (2013). Comparison of a SiO 2–CaO–ZnO–SrO glass polyalkenoate cement to commercial dental materials: ion release, biocompatibility and antibacterial properties. J. Mater. Sci. 24, 2255–2264. 10.1007/s10856-013-4974-6 23793491

[B178] Wren A. Cummins N. M. Coughlan A. Towler M. (2010). The effect of adding organic polymers on the handling properties, strengths and bioactivity of a Ca–Sr–Zn–Si glass polyalkenoate cement. J. Mater. Sci. 45, 3554–3562. 10.1007/s10853-010-4398-3

[B179] Wright A. C. Dalba G. Rocca F. Vedishcheva N. M. (2010). Borate versus silicate glasses: why are they so different? Phys. Chem. Glasses-Eur. J. Glass Sci. Technol. Part B, 51, 233–265.

[B180] Wu C. Fan W. Zhu Y. Gelinsky M. Chang J. Cuniberti G.. (2011). Multifunctional magnetic mesoporous bioactive glass scaffolds with a hierarchical pore structure. Acta Biomater. 7, 3563–3572. 10.1016/j.actbio.2011.06.028 21745610

[B181] Wu C. Ramaswamy Y. Kwik D. Zreiqat H. (2007). The effect of strontium incorporation into CaSiO3 ceramics on their physical and biological properties. Biomaterials 28, 3171–3181. 10.1016/j.biomaterials.2007.04.002 17445881

[B182] Wu C. Zhang Y. Zhu Y. Friis T. Xiao Y. (2010). Structure–property relationships of silk-modified mesoporous bioglass scaffolds. Biomaterials 31, 3429–3438. 10.1016/j.biomaterials.2010.01.061 20122721

[B183] Wu C. Zhou Y. Lin C. Chang J. Xiao Y. (2012). Strontium-containing mesoporous bioactive glass scaffolds with improved osteogenic/cementogenic differentiation of periodontal ligament cells for periodontal tissue engineering. Acta Biomater. 8, 3805–3815. 10.1016/j.actbio.2012.06.023 22750735

[B184] Wu C. Zhou Y. Xu M. Han P. Chen L. Chang J.. (2013). Copper-containing mesoporous bioactive glass scaffolds with multifunctional properties of angiogenesis capacity, osteostimulation and antibacterial activity. Biomaterials 34, 422–433. 10.1016/j.biomaterials.2012.09.066 23083929

[B185] Xiang Y. Du J. (2011). Effect of strontium substitution on the structure of 45S5 bioglasses. Chem. Mater. 23, 2703–2717. 10.1021/cm102889q

[B186] Xiang Y. Du J. Skinner L. B. Benmore C. J. Wren A. W. Boyd D. J. (2013). Structure and diffusion of ZnO–SrO–CaO–Na2O–SiO2 bioactive glasses: a combined high energy X-ray diffraction and molecular dynamics simulations study. RSC Adv. 3, 5966–5978. 10.1039/c3ra23231j

[B187] Xuereb M. Camilleri J. Attard N. J. (2015). Systematic review of current dental implant coating materials and novel coating techniques. Int. J. Prosthodont. 28, 51–9. 10.11607/ijp.4124 25588174

[B188] Yang F. Yang D. Tu J. Zheng Q. Cai L. Wang L. (2011). Strontium enhances osteogenic differentiation of mesenchymal stem cells and *in vivo* bone formation by activating Wnt/catenin signaling. Stem Cells 29, 981–991. 10.1002/stem.646 21563277

[B189] Yin H. Yang C. Gao Y. Wang C. Li M. Guo H. (2018). Fabrication and characterization of strontium-doped borate-based bioactive glass scaffolds for bone tissue engineering. J. Alloys Comp. 743, 564–569. 10.1016/j.jallcom.2018.01.099

[B190] Ylanen H. O. (2011). Bioactive glasses: materials, properties, and applications. Addit. Manufactur. 24, 647–657. 10.1533/9780857093318

[B191] Yu T. Ye J. Wang Y. (2009). Preparation and characterization of a novel strontium-containing calcium phosphate cement with the two-step hydration process. Acta Biomater. 5, 2717–2727. 10.1016/j.actbio.2009.03.015 19380262

[B192] Zhang J. Zhao S. Zhu Y. Huang Y. Zhu M. Tao C.. (2014b). Three-dimensional printing of strontium-containing mesoporous bioactive glass scaffolds for bone regeneration. Acta Biomater. 10, 2269–2281. 10.1016/j.actbio.2014.01.001 24412143

[B193] Zhang L. Tan P. Y. Chow C. L. Lim C. K. Tan O. K. Tse M. S. (2014c). Antibacterial activities of mechanochemically synthesized perovskite strontium titanate ferrite metal oxide. Colloids Surfaces A 456, 169–175. 10.1016/j.colsurfa.2014.05.032

[B194] Zhang Q. Chen X. Geng S. Wei L. Miron R. J. Zhao Y.. (2017). Nanogel-based scaffolds fabricated for bone regeneration with mesoporous bioactive glass and strontium: *in vitro* and *in vivo* characterization. J. Biomed. Mat. Res. A 105, 1175–1183. 10.1002/jbm.a.35980 27998017

[B195] Zhang W. Zhao F. Huang D. Fu X. Li X. Chen X. (2016). Strontium-substituted submicrometer bioactive glasses modulate macrophage responses for improved bone regeneration. ACS Appl. Mater. Interfaces 8, 30747–30758. 10.1021/acsami.6b10378 27779382

[B196] Zhang X. Lianghao W. Dejian L. Rongguang A. Chen F. Bin Y. (2017). Three-dimensional printing of strontium-containing mesoporous bioactive glass scaffolds with varied macropore morphologies: an *in vitro* cytological experiment. Chin. J. Tissue Eng. Res. 21, 2858–2863. 10.3969/j.issn.2095-4344.2017.18.012

[B197] Zhang Y. Cui X. Zhao S. Wang H. Rahaman M. N. Liu Z.. (2015). Evaluation of injectable strontium-containing borate bioactive glass cement with enhanced osteogenic capacity in a critical-sized rabbit femoral condyle defect model. ACS Appl. Mater. Interfaces 7, 2393–2403. 10.1021/am507008z 25591177

[B198] Zhang Y. Wei L. Chang J. Miron R. J. Shi B. Yi S. (2013). Strontium-incorporated mesoporous bioactive glass scaffolds stimulating *in vitro* proliferation and differentiation of bone marrow stromal cells and in vivo regeneration of osteoporotic bone defects. J. Mater. Chem. B 1, 5711–5722. 10.1039/C3TB21047B 32261194

[B199] Zhang Y. Wei L. Wu C. Miron R. J. (2014a). Periodontal regeneration using strontium-loaded mesoporous bioactive glass scaffolds in osteoporotic rats. PLoS ONE 9:e104527. 10.1371/journal.pone.0104527 25116811 PMC4130544

[B200] Zhao F. Lei B. Li X. Mo Y. Wang R. Chen D.. (2018). Promoting *in vivo* early angiogenesis with sub-micrometer strontium-contained bioactive microspheres through modulating macrophage phenotypes. Biomaterials 178, 36–47. 10.1016/j.biomaterials.2018.06.004 29908343

[B201] Zhao S. Zhang J. Zhu M. Zhang Y. Liu Z. Tao C.. (2015). Three-dimensional printed strontium-containing mesoporous bioactive glass scaffolds for repairing rat critical-sized calvarial defects. Acta Biomater. 12, 270–280. 10.1016/j.actbio.2014.10.015 25449915

[B202] Zhu Y. Li X. Yang J. Wang S. Gao H. Hanagata N. (2011). Composition–structure–property relationships of the CaO–M x O y–SiO 2–P 2 O 5 (M = Zr, Mg, Sr) mesoporous bioactive glass (MBG) scaffolds. J. Mater. Chem. 21, 9208–9218. 10.1039/c1jm10838g

